# Efficacy and safety of ascending dosages of albendazole against *Trichuris trichiura* in preschool-aged children, school-aged children and adults: A multi-cohort randomized controlled trial

**DOI:** 10.1016/j.eclinm.2020.100335

**Published:** 2020-05-05

**Authors:** Chandni Patel, Jean T. Coulibaly, Jessica D. Schulz, Yves N'Gbesso, Jan Hattendorf, Jennifer Keiser

**Affiliations:** a Swiss Tropical and Public Health Institute, Basel, Switzerland; b University of Basel, Basel, Switzerland; c Unité de Formation et de Recherche Biosciences, Université Félix Houphouët-Boigny, Abidjan, Côte d'Ivoire and Centre Suisse de Recherches Scientifiques en Côte d'Ivoire, Abidjan, Côte d'Ivoire; d Department de Agboville, Centre de Santé Urbain d'Azaguié, Côte d'Ivoire

**Keywords:** Soil-transmitted helminths, Albendazole, Trichuis trichiura, Dose-finding

## Abstract

**Background:**

The efficacy of the widely used albendazole against the soil-transmitted helminth *Trichuris trichiura* is limited; yet optimal doses, which may provide increased efficacy, have not been thoroughly investigated to date.

**Methods:**

A randomized-controlled trial was conducted in Côte d'Ivoire with preschool-aged children (PSAC), school-aged children (SAC), and adults infected with *T. trichiura*. Participants were randomly assigned (1:1:1:1) using computer-generated randomization. PSAC were randomized to 200 mg, 400 mg, 600 mg of albendazole or placebo. SAC and adults were randomized to 400 mg, 600 mg, 800 mg of albendazole or placebo. The primary outcome was cure rates (CRs) against trichuriasis. Secondary outcomes were *T. trichiura* egg reduction rates (ERRs), safety, CRs and ERRs against other soil-transmitted helminths. Outcome assessors and the trial statistician were blinded. Trial registration at ClinicalTrial.gov: NCT03527745.

**Findings:**

111 PSAC, 180 SAC, and 42 adults were randomized and 86, 172, and 35 provided follow-up stool samples, respectively. The highest observed CR among PSAC was 27·8% (95% CI: 9·7%–53·5%) in the 600 mg albendazole treatment arm. The most efficacious arm for SAC was 600 mg of albendazole showing a CR of 25·6% (95% CI: 13·5%–41·2%), and for adults it was 400 mg of albendazole with a CR of 55·6% (95% CI: 21·2%–86·3%). CRs and ERRs did not differ significantly among treatment arms and flat dose-responses were observed. 17·9% and 0·4% of participants reported any adverse event at 3 and 24 h follow-up, respectively.

**Interpretation:**

Albendazole shows low efficacy against *T. trichiura* in all populations and doses studied, though findings for PSAC and adults should be carefully interpreted as recruitment targets were not met. New drugs, treatment regimens, and combinations are needed in the management of *T. trichiura* infections.

**Funding:**

Bill and Melinda Gates Foundation.

**Research in context**

***Evidence before this study***

A literature review was conducted in PubMed searching for “Trichuris” and “albendazole” from inception to January 1, 2020. Different dosages and regimens of albendazole have been tested against *T. trichiura*; however, a thorough dose-finding study has not been done to date for preschool-aged children, school-aged children, and adults.

***Added value of this study***

This is the first multi-cohort, randomized-controlled trial conducted to determine the optimal dose of albendazole in several age groups targeted by treatment recommendations of the WHO. Based on the results of this trial in Côte d'Ivoire, it can be concluded that the current recommended dose of 400 mg of albendazole is not efficacious and higher doses do not provide any added benefit.

***Implications of all the available evidence***

Treatment with a single dose of the benzimidazoles annually or biannually in areas of STH prevalence ≥20% is the current key recommendations for the control of soil-transmitted helminths; however, efficacy of the drugs against trichuriasis is very low. Evidence from this trial confirms that albendazole, even at higher doses, is insufficient for treating *T. trichiura* infections; and, therefore novel treatments or combination therapy should be considered as part of control efforts to control and ultimately eliminate STHs.

Alt-text: Unlabelled box

## 1 Introduction

Almost a quarter of the world's population is infected with soil-transmitted helminths (STHs): *Trichuris trichiura* (whipworm)*, Ascaris lumbricoides* (roundworm)*,* and *Ancylostoma duodenale* or *Necator americanus* (hookworms) [1]. STH infections account for a burden of over 1·9 million disability-adjusted life-years (DALYs) per year with trichuriasis accounting for 213 thousand DALYs [2]. Greatest at risk for infection are those living without access to potable water and living with inadequate sanitation in tropical climates [3], [4], [5]. Heavy intensity infections may lead to diarrhea, abdominal pain, inflammation, obstruction and, if untreated, nutrition and immune system impairment [3,6].

Preventive chemotherapy (PC), recommended by the World Health Organization (WHO) is the periodic administration of anthelmintic drugs through mass drug administration (MDA) campaigns [1]. PC has been successful in reducing the number of STH infections and reducing the burden of disease (especially moderate and heavy infections) by averting an estimated 61 thousand DALYs from 2010 to 2015 [7,8]. Current recommendations for MDA of first-line treatment include monotherapy of 400 mg of albendazole or 500 mg of mebendazole once or twice a year targeting children, girls and women of reproductive age, and women after the first trimester of pregnancy in settings where STH prevalence is ≥20% [1,9]. However, albendazole and mebendazole show limited efficacy against *T. trichiura* (cure rates of 30% and 42%, respectively) [9,10].

To date, the optimal dose for albendazole has not been determined and 400 mg is the standard dose regardless of age and/or weight [1,11]. Though a driver of efficacy has not been identified, hypothetically, a higher dose may be needed for school-aged children (SAC) and adults. Different doses of albendazole have been tested for efficacy against *T. trichiura*, but a formal dose-response relationship has not been conducted [12]. The objective of this trial was for the first time to determine the efficacy and safety of ascending doses of albendazole against *T. trichiura* in three population cohorts (preschool-aged children (PSAC), SAC, and adults).

## 2 Methods

### 2.1 Study design

A phase two, parallel, randomized, placebo-controlled, dose-finding trial was conducted in seven villages near the town of Azaguié in the Agboville department of southern Côte d'Ivoire from October 23, 2018 to January 12, 2019. PSAC 2–5 years of age, SAC 6–12 years of age, and adults over the age of 21 years were invited to participate in the trial. It was decided not to include community members aged 13–20 years for two reasons: no differences between teenagers and adults were expected and teenage community members are the most transient population in remote villages based on our own previous experience. Ethical clearance was obtained from the Comité National d’Éthique des Sciences de la Vie et de la Santé (July 3, 2018, reference No. 089-18//MSHP/CNESVS-km), and the Ethics Committee of Northwestern and Central Switzerland (July 20, 2018, Nr 2018-00545).

### 2.2 Participants

Prior to enrolment, an information session explaining the purpose, procedure, benefits, and risks of the trial was conducted in each of the seven villages for all community members. Written informed consent was obtained from all participants and/or parents and guardians of the children after they had attended the information session. Additionally, SAC provided written assent.

PSAC, SAC, and adults were eligible if they provided two stool samples and were positive for *T. trichiura*. Only PSAC with *T. trichiura* infection intensities ≥60 eggs per gram of stool (EPG) and SAC and adults with *T. trichiura* infection intensities ≥100 EPG were included in the trial. Excluded from the trial were those with acute or uncontrolled systemic illnesses (e.g., severe anemia defined as hemoglobin <8.0 g/dl, infection, clinical malaria) as assessed by a medical doctor upon initial clinical assessment and liver function tests, those who had received anthelmintic within the previous 4 weeks, those who were allergic to benzimidazoles, and pregnant or breastfeeding women.

### 2.3 Randomization and masking

Study participants eligible for treatment were randomly assigned to one of the treatment arms using a computer-generated, stratified block randomization code. A random allocation sequence with varying random blocks of 4 and 8 stratified by 2 levels of baseline infection intensity (light and moderate/heavy *T. trichiura*) according to WHO guidelines was provided by a statistician not involved in recruitment or data collection [13]. Since so few moderate and high infections are present in the setting, it was decided to combine these two levels of intensity into one stratum. PSAC were 1:1:1:1 randomized to placebo or albendazole at 200 mg, 400 mg, or 600 mg. SAC and adults were 1:1:1:1 randomized to placebo or albendazole at 400 mg, 600 mg, or 800 mg. Study-site investigators were aware of the study group assignment, whereas outcome assessors and the trial statistician were masked to group assignment. Participants might have recognized the study group assignment due to the number of tablets administered.

### 2.4 Procedures

Children and adults provided two stool samples collected on two different days after providing name, age, and sex in a village-wide census. Duplicate Kato-Katz thick smears (41·7 mg each) were prepared from each sample and examined for *T. trichiura, Ascaris lumbricoides*, and hookworm eggs [14]. Egg counts were recorded by experienced technicians and classified as light, moderate, or heavy [14]. An independent quality control was conducted for 10% of the slides prepared with results considered correct if: (i) no difference in presence/absence of *T. trichiura, A. lumbricoides,* and hookworm; (ii) egg counts were +/−10 eggs for counts ≤100 eggs or +/−20% for counts >100 eggs (for each species separately) [15].

Before treatment, eligible children and adults were examined physically and questioned for clinical symptoms by a trial physician. Height using a stadiometer, weight using a scale, and temperature using an ear thermometer were collected by trial nurses. In addition, venous blood samples were taken at baseline to assess organ toxicity, complete blood count, and hepatic/renal function, while capillary blood samples using the finger-prick method were used to measure hemoglobin levels (HemoCue 301) and diagnose malaria through a rapid test (ICT Malaria P.f. Antigen Test, ICT Diagnostics).

On the day of treatment, enrolled participants were given potable water and a high-fat breakfast before receiving either albendazole or placebo orally with water. Active adverse event assessment using a questionnaire was conducted at 3 h, 24 h, and 14–21 days after treatment. Participants were asked about the occurrence and intensity of headache, abdominal pain, nausea, vomiting, diarrhea, itching, allergic reactions, and any other symptom; moreover, temperature was taken to assess fever. Between 14 and 21 days post-treatment, treatment efficacy was assessed by collecting and examining two additional stool samples as described above. At the end of the trial, all participants and excluded children/adults remaining positive for any helminth infection were treated with 400 mg of albendazole in accordance with WHO guidelines [1].

### 2.5 Outcomes

The primary outcome is cure rate (CR) against *T. trichiura* at 14–21 days post-treatment. Secondary outcomes include egg reduction rate (ERR) against *T. trichiura*, CRs and ERRs against *A. lumbricoides* and hookworm, and drug safety. Drug safety was assessed at 3 h, 24 h, and 14–21 days after treatment.

### 2.6 Sample size

The main aim of this trial is to determine the dose-response relationship of albendazole against *T. trichiura*. A series of simulations were carried out to determine the required sample size. We assumed a true CR of 5%, 10%, 20%, 30%, and 40% against *T. trichiura* and a loss to follow-up of 5%. We estimated that enrolling 40 participants per arm will be sufficient to predict the dose response curve with a precision of about 10% points [16]. See supplementary material for details in sample size determination.

### 2.7 Statistical analyses

All data were collected on paper forms and entered twice into a database (Access 2010, Microsoft), cross-checked using the Data Compare utility of EpiInfo, version 3.5.4 (Centers for Disease Control and Prevention, Atlanta, GA, SA), and any discrepancies corrected by consulting the hard copy. Data management and descriptive results were done in Stata, version 15 (StataCorp, College Station, TX, USA), and the estimation of the dose-response curve was performed in R, version 3.5.1 (www.r-project.org).

An available-case analysis was performed according to the intention-to-treat principle using a full analysis set of all randomized participants providing any follow-up data. A per protocol analysis was planned according to the protocol. Since there were no protocol deviations the per protocol population was identical to the available case population. CRs were calculated as the percentage of egg-positive participants at baseline who become egg-negative after treatment. EPG was assessed by calculating the mean egg counts from the quadruplicate Kato-Katz thick smears and multiplying this number by a factor of 24. The ERR was calculated with the following formula:

$$ERR=1-\frac{\frac{1}{n}e^{\sum \log\left(EPG_{follow-up}+1\right)}-1}{\frac{1}{n}\;e^{\;\sum \log\left(EPG_{baseline}+1\right)}-1}$$

Geometric mean egg counts were calculated for the different treatment arms before and after treatment to assess the corresponding ERRs. Bootstrap resampling method with 5000 replicates was used to calculate 95% confidence intervals (CIs) for ERRs point estimates. ERRs were not calculated when too few infections were observed; moreover, 95% CIs were not calculated in cases of too small sample sizes for other helminth infections.

The hyperbolic *E*_max_ dose-response model was fitted using the DoseRange packages version 0.9–17 with the coefficients and the variance-covariance matrix estimates from a logistic regression. The analysis code is similar to the one used for the sample size simulations (Supplementary Material). The half–maximal–effect dose (ED_50_) was estimated as half of the maximal placebo-adjusted effect on logit scale and afterwards back-transformed to probability (i.e., cure rate) scale.

Adverse events were descriptively evaluated as the number and proportion of participants reporting adverse events before and after treatment. The trial is registered at ClinicalTrials.gov (NCT03527745).

### 2.8 Role of the funding source

The funder of the study had no role in the study design, data collection, data analysis, data interpretation, or writing of the report. The corresponding author had full access to all the data in the study and had final responsibility for the decision to submit for publication.

## 3 Results

### 3.1 Baseline characteristics

**Fig. 1:**
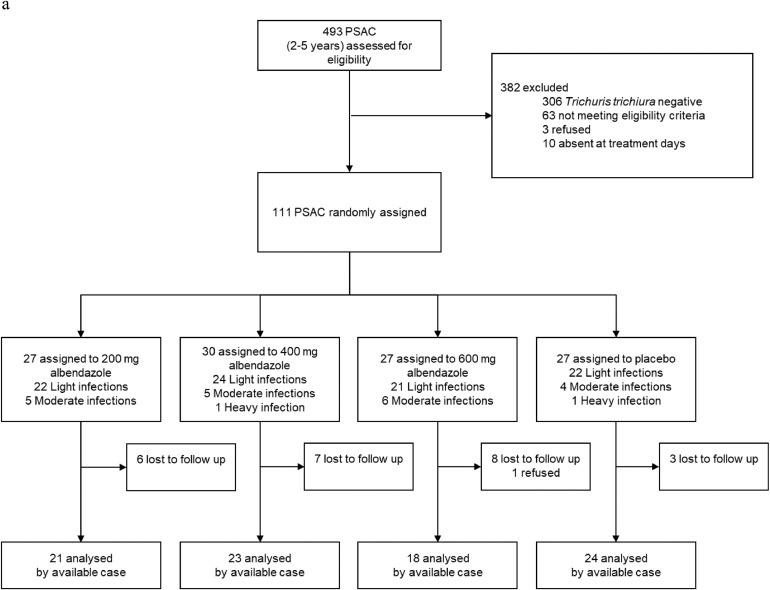

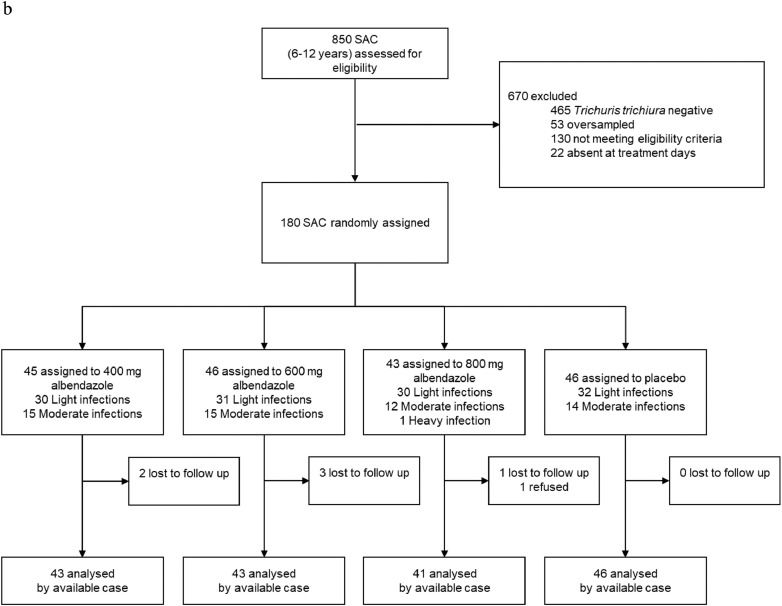

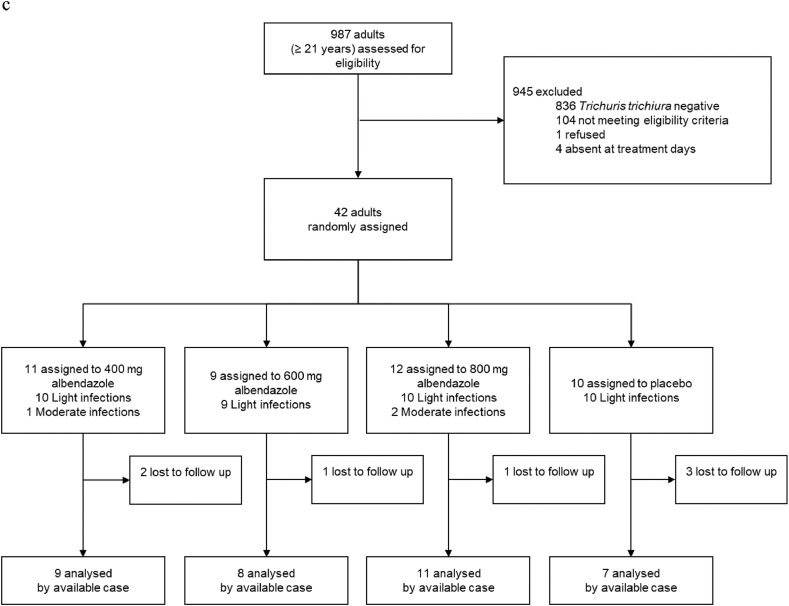
a: PSAC participant flow-chart. Abbreviations: EPG, eggs per gram; PSAC, preschool-aged children. b: SAC participant flow-chart. Abbreviations: EPG, eggs per gram; SAC, preschool-aged children. c: Adults participant flow-chart. Abbreviations: EPG, eggs per gram.

Participants were recruited for the trial between October 23, 2018 and December 1, 2018. Despite an intensive screening effort, the targeted sample size (160 participants in each age cohort) was not reached for PSAC (69%) and adults (26%). A total of 2330 (493 PSAC, 850 SAC, and 987 adults) were screened for eligibility. Of those screened, 137 PSAC, 273 SAC, and 56 adults were invited for clinical examination, while 1607 were negative for *T. trichiura* and 50 PSAC, 112 SAC, and 95 adults had too low infection intensities. Of those invited to clinical examination, 26 PSAC, 40 SAC, and 14 adults either refused participation, were excluded based on eligibility criteria, or absent on treatment day. 53 eligible SAC children were oversampled and not enrolled in the trial. A total of 111 PSAC, 180 SAC, and 42 adults were randomly assigned to one of four treatment arms (Fig. 1a–c). At follow-up, 25 PSAC (22·5%), 7 SAC (3·9%), and 7 adults (16·7%) did not provide stool samples or were absent.

Baseline demographic and parasitological characteristics of PSAC, SAC, and adults included in the trial are presented in Table 1. Age, weight, height, and infection intensity were balanced across arms within the PSAC and SAC cohorts. In PSAC and adult cohorts, there were more female than male participants across all arms. Among SAC, there were more females in the placebo group in comparison to those receiving 400 mg of albendazole (25 vs 15); all other arms were balanced in terms of sex. The majority of infections were of light intensity (ranging 67–81% across arms) in all three age cohorts. In total, 22 PSAC, 57 SAC, and 3 adults had moderate or heavy intensity infections. There were very few co-infections with *A. lumbricoides* and/or hookworm: 20 PSAC, 54 SAC, and 6 adults were infected with *A. lumbricoides* and 1 PSAC, 7 SAC, and 2 adults were infected with hookworm.

**Table 1:** Baseline characteristics of participants. Numbers represent N (%) unless otherwise stated. Abbreviations: ALB, albendazole; EPG, eggs per gram; IQR, interquartile range; PLAC, placebo; PSAC, preschool-aged children; SAC, school-aged children; SD, standard deviation.

	PSAC				SAC				Adults			
	ALB 200 mg (*n* = 27)	ALB 400 mg (*n* = 30)	ALB 600 mg (*n* = 27)	PLAC (*n* = 27)	ALB 400 mg (*n* = 45)	ALB 600 mg (*n* = 46)	ALB 800 mg (*n* = 43)	PLAC (*n* = 46)	ALB 400 mg (*n* = 11)	ALB 600 mg (*n* = 9)	ALB 800 mg (*n* = 12)	PLAC (*n* = 10)
Mean (SD) age (years)	4·0 (0·9)	3·8 (1·1)	4·0 (0·9)	4·1 (0·9)	8·9 (1·9)	8·7 (2·0)	8·8 (1·8)	8·9 (1·9)	40·5 (11·9)	36·6 (12·2)	44·8 (15·9)	44·4 (12·3)
Females	18 (67%)	16 (53%)	18 (67%)	16 (59%)	15 (33%)	19 (41%)	17 (40%)	25 (54%)	9 (82%)	7 (78%)	8 (67%)	6 (60%)
Mean (SD) weight (kg)	15·1 (2·9)	14·8 (2·2)	15·1 (2·3)	14·6 (2·5)	26·2 (6·5)	24·1 (5·7)	24·3 (4·1)	25·3 (6·6)	54·0 (7·6)	60·5 (8·9)	57·0 (6·0)	57·4 (9·7)
Mean (SD) height (cm)	97·2 (9·5)	99·2 (10·4)	97·6 (10·1)	97·5 (10·7)	126·8 (10·5)	123·7 (11·4)	125·0 (8·8)	125·8 (12·2)	157·5 (5·3)	158·8 (5·8)	158·5 (8·3)	160·1 (8·5)
***Trichuris trichiura***												
Median EPG [IQR]	264 [96–558]	210 [144–510]	228 [150–726]	216 [120–438]	426 [192–1266]	507 [186–1134]	414 [162–1116]	387 [222–1068]	168 [114–504]	156 [114–288]	195 [150–402]	138 [114–270]
EPG geometric mean	303·4	303·9	328·5	371·7	538·6	510·3	501·1	521·4	257·4	204·8	272·8	173·0
Infection intensity												
Light (1–999 EPG)	22 (81%)	24 (80%)	21 (78%)	22 (81%)	30 (67%)	31 (67%)	30 (70%)	32 (70%)	10 (91%)	9 (100%)	10 (83%)	10 (100%)
Moderate (1000–9999 EPG)	5 (19%)	5 (17%)	6 (22%)	4 (15%)	15 (33%)	15 (33%)	12 (28%)	14 (30%)	1 (9%)	0 (0%)	2 (17%)	0 (0%)
Heavy (≥10,000 EPG)	0 (0%)	1 (3%)	0 (0%)	1 (4%)	0 (0%)	0 (0%)	1 (2%)	0 (0%)	0 (0%)	0 (0%)	0 (0%)	0 (0%)
**Hookworm**												
Infected children	0	1	0	0	1	2	3	1	1	0	0	1
***Ascaris lumbricoides***												
Infected children	8	5	3	4	18	10	13	13	3	0	3	0
Median EPG [IQR]	7929 [3123–10,395]	2118 [1428–6144]	1938 [30–10,530]	6216 [723–12,987]	5526 [690–17,160]	14,823 [1962–25,050]	7668 [2520–14,952]	528 [210–13,122]	13,500 [3906–18,954]		1068 [168–12,600]	
EPG geometric mean	3190·4	2163·3	857·6	2606·3	2752·7	4418·1	3241·1	1078·3	9998·5		1314·5	
Infection intensity												
Light (1–4999 EPG)	2 (25%)	3 (60%)	2 (67%)	2 (50%)	8 (44%)	4 (40%)	6 (46%)	8 (62%)	1	0	2	0
Moderate (5000–49,999 EPG)	6 (75%)	2 (40%)	1 (33%)	2 (50%)	9 (50%)	6 (60%)	7 (54%)	5 (38%)	2	0	1	0
Heavy (≥50,000 EPG)	0 (0%)	0 (0%)	0 (0%)	0 (0%)	1 (6%)	0 (0%)	0 (0%)	0 (0%)	0	0	0	0

### 3.2 Efficacy against *T. trichiura*

**Fig. 2:**
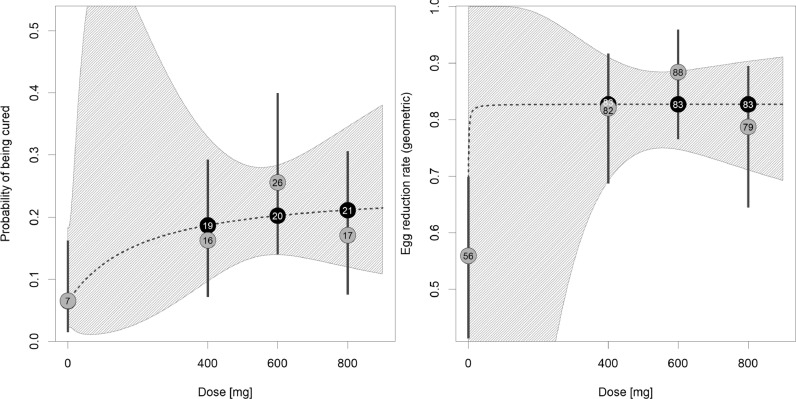
CRs and ERRs predicted by the hyperbolic *E*_max_ model. Dotted lines represent the dose-response curve and the hatched area corresponds to the 95% confidence band. White numbers present the predicted CRs and ERRs for the investigated doses. Gray circles with black numbers represent the observed dose group CRs and the gray vertical lines correspond to the 95% confidence intervals. Predicted and observed estimates are similar in the placebo group; therefore, only one number is provided.

CRs and ERRs of each arm against *T. trichiura* are shown in Table 2 for SAC, while results for PSAC and adults can be found in Supplementary Table S1. The *E*_max_ model predicted a maximal CR (*E*_max_) of 24·7% and an ED_50_ at 118 mg. This predicted dose–response curve showed a plateau at approximately 500 mg (Fig. 2) with predicted CRs for the investigated doses of 6·5% (95% CI: 2·1%–18·3%) in the placebo group and 18·7% (95% CI: 9·7%–32·9%), 20·2% (95% CI: 13·9%–28·4%), and 21·1% (95% CI: 12·0%–34·6%) at 400 mg, 600 mg, and 800 mg, respectively. The observed CRs were close to the predicted values, except at 600 mg where the observed CR was 5.4%–points higher (25·6%, 95% CI: 13·5%–41·2%). Observed ERRs plateaued already at the first investigated dose (400 mg, ERR: 82·0%, 95% CI: 67·8%–90·5%).

**Table 2:** Observed and predicted cure rates and egg reduction rates against *Trichuris trichiura* of SAC at 3 weeks follow-up. Abbreviations: ALB, albendazole; CI, confidence interval; CR, cure rate; EPG, eggs per gram; ERR, egg reduction rate; PLAC, placebo; SAC, school-aged children.

	ALB 400 mg	ALB 600 mg	ALB 800 mg	PLAC
Positive before treatment (n)	43	43	41	46
Cured after treatment (n)	7	11	7	3
Observed CR [95% CI]	16·3 [6·8, 30·7]	25·6 [13·5, 41·2]	17·1 [7·2, 32·1]	6·5 [1·4, 17·9]
Predicted CR [95% CI]	18·7 [9·7, 32·9]	20·2 [13·9, 28·4]	21·1 [12·0, 34·6]	6·5 [2·1, 18·3]
EPG geometric mean				
Baseline	542·3	517·3	507·9	521·9
3 weeks follow-up	97·4	59·9	108·1	230·1
Observed ERR [95% CI]	82·0 [67·8, 90·5]	88·4 [74·8, 94·9]	78·7 [60·5, 89·0]	55·9 [26·1, 75·2]
EPG arithmetic mean				
Baseline	1094·4	955·3	1247·4	1094·0
3 weeks follow-up	545·2	687·1	893·6	1009·8
Observed ERR [95% CI]	50·2 [21·5, 67·9]	28·1 [−49·5, 69·6]	28·4 [2·6, 54·3]	7·7 [−46·0, 43·1]

Observed CRs among PSAC were similar across all arms ranging from 9·5% (95% CI: 1·2%–30·4%) to 27·8% (95% CI: 9·7%–53·5%). The corresponding geometric ERRs found were also similar across treatment arms ranging from 63·8% in the 200 mg albendazole arm to 88·5% in the 600 mg albendazole arm. Observed CRs for adults were 55·6% (95% CI: 21·2%–86·3%), 50·0% (95% CI: 15·7%–84·3%), 27·3% (95% CI: 6·0%–61·0%), 14·3(95% CI: 0·4%–57·9%) for albendazole at 400 mg, 600 mg, 800 mg, and placebo, respectively. The geometric ERRs ranged from 85·2% (placebo arm) to 97·7% (400 mg of albendazole arm).

Supplementary Table S2 presents the proportions of participants cured within each treatment arm by sex.

### 3.3 Efficacy against hookworm and *A. lumbricoides*

Data on CRs and ERRs against other helminth infections for PSAC, SAC, and adults are presented in Supplementary Table S3. For hookworm, 7 out of the 10 infections with follow-up data were cured with any dose of albendazole across all treatment arms in all cohorts.

For roundworm, CRs ranged between 66·7%–100% for PSAC given albendazole and 0% for PSAC given placebo. For SAC, CRs ranged from 84·6%–100% in active treatment arms and 38·5% in the placebo arm. All adults with *A. lumbricoides* infections were cured receiving either 400 mg or 800 mg of albendazole. Geometric and arithmetic ERRs had similarly large ranges for PSAC and SAC for all active treatment groups (placebo groups were considerably lower), and 100% for the adults cured by 400 mg or 800 mg of albendazole.

### 3.4 Safety

At baseline 102 PSAC, 172 SAC, and 42 adults were questioned for symptoms. A total of 84 (26·6%) participants reported mild symptoms at baseline, such as abdominal pain (11·4%), headache (11·1%), itching (5·4%), and diarrhea (4·4%).

104 PSAC, 175 SAC, and 41 adults were interviewed at 3 h post-treatment for adverse events and 86 PSAC, 154 SAC, and 32 adults were interviewed at 24 h post-treatment for adverse events. After treatment the proportion of reporting any adverse events was 17·9% and 0·4% at 3 and 24 h follow-up, respectively. Numbers of participants reporting each adverse event is reported in Table 3.

**Table 3:** Number of participants experiencing adverse events. Abbreviations: ALB, albendazole; CI, confidence interval; CR, cure rate; EPG, eggs per gram; ERR, egg reduction rate; PLAC, placebo; PSAC, preschool-aged children; SAC, school-aged children.

	PSAC				SAC				Adults			
Adverse Event	ALB 200 mg	ALB 400 mg	ALB 600 mg	PLAC	ALB 400 mg	ALB 600 mg	ALB 800 mg	PLAC	ALB 400 mg	ALB 600 mg	ALB 800 mg	PLAC
**Before treatment**												
Headache	0/24 (0·0)	2/26 (7·7)	0/26 (0·0)	1/26 (3·9)	5/41 (12·2)	3/45 (6·7)	5/42 (11·9)	6/44 (13·6)	6/11 (54·6)	2/9 (22·2)	3/12 (25·0)	2/10 (20·0)
Abdominal pain	0/24 (0·0)	1/26 (3·9)	2/26 (7·7)	1/26 (3·9)	8/41 (19·5)	5/45 (11·1)	10/42 (23·8)	4/44 (9·1)	0/11 (0·0)	1/9 (1·1)	1/12 (8·3)	3/10 (30·0)
Nausea	0/24 (0·0)	0/26 (0·0)	0/26 (0·0)	0/26 (0·0)	1/41 (2·4)	0/45 (0·0)	0/42 (0·0)	1/44 (2·3)	1/11 (9·1)	1/9 (1·1)	1/12 (8·3)	0/10 (0·0)
Vomiting	0/24 (0·0)	0/26 (0·0)	0/26 (0·0)	0/26 (0·0)	0/41 (0·0)	0/45 (0·0)	2/42 (4·8)	1/44 (2·3)	0/11 (0·0)	0/9 (0·0)	0/12 (0·0)	0/10 (0·0)
Diarrhea	2/24 (8·3)	1/26 (3·9)	2/26 (7·7)	1/26 (3·9)	0/41 (0·0)	2/45 (4·4)	2/42 (4·8)	4/44 (4·6)	0/11 (0·0)	0/9 (0·0)	0/12 (0·0)	0/10 (0·0)
Itching	0/24 (0·0)	0/26 (0·0)	1/26 (3·9)	1/26 (3·9)	4/41 (9·8)	1/45 (2·2)	3/42 (7·1)	0/44 (0·0)	2/11 (18·2)	1/9 (1·1)	3/12 (25·0)	1/10 (10·0)
**3 hr post treatment**												
Headache	2/25 (8·0)	3/27 (11·1)	0/26 (0·0)	1/26 (3·9)	1/42 (2·4)	3/45 (6·7)	5/42 (11·9)	3/46 (6·5)	3/11 (27·3)	2/9 (22·2)	2/11 (18·2)	1/10 (10·0)
Abdominal pain	0/25 (0·0)	1/27 (3·7)	2/26 (7·7)	1/26 (3·9)	4/43 (9·3)	3/45 (6·7)	7/42 (16·7)	4/46 (8·7)	3/11 (27·3)	1/9 (11·1)	0/11 (0·0)	2/10 (20·0)
Nausea	0/25 (0·0)	0/27 (0·0)	0/26 (0·0)	0/26 (0·0)	0/43 (0·0)	0/45 (0·0)	0/41 (0·0)	0/46 (0·0)	1/11 (9·1)	0/9 (0·0)	0/11 (0·0)	0/10 (0·0)
Vomiting	0/25 (0·0)	0/27 (0·0)	0/26 (0·0)	0/26 (0·0)	0/43 (0·0)	0/45 (0·0)	0/42 (0·0)	1/46 (2·2)	0/11 (0·0)	0/9 (0·0)	0/11 (0·0)	0/10 (0·0)
Diarrhea	0/25 (0·0)	2/27 (7·4)	1/26 (3·9)	1/26 (3·9)	3/43 (7·0)	1/45 (2·2)	0/42 (0·0)	2/46 (4·4)	0/11 (0·0)	0/9 (0·0)	0/11 (0·0)	0/10 (0·0)
Itching	0/25 (0·0)	0/27 (0·0)	0/26 (0·0)	0/26 (0·0)	1/43 (2·3)	1/45 (2·2)	0/42 (0·0)	0/46 (0·0)	0/11 (0·0)	0/9 (0·0)	1/9 (11·1)	0/10 (0·0)
Serious adverse event	1/25 (4·0)	0/27 (0·0)	0/26 (0·0)	0/26 (0·0)	0/43 (0·0)	0/45 (0·0)	0/42 (0·0)	0/46 (0·0)	0/11 (0·0)	0/9 (0·0)	0/11 (0·0)	0/10 (0·0)
**24 hr post treatment**												
Headache	0/21 (0·0)	0/25 (0·0)	0/21 (0·0)	0/19 (0·0)	0/37 (0·0)	0/41 (0·0)	0/39 (0·0)	0/37 (0·0)	0/10 (0·0)	0/7 (0·0)	0/9 (0·0)	0/6 (0·0)
Abdominal pain	0/21 (0·0)	0/25 (0·0)	0/21 (0·0)	0/19 (0·0)	0/37 (0·0)	0/41 (0·0)	0/39 (0·0)	0/37 (0·0)	0/10 (0·0)	0/7 (0·0)	0/9 (0·0)	0/6 (0·0)
Nausea	0/21 (0·0)	0/25 (0·0)	0/21 (0·0)	0/19 (0·0)	0/37 (0·0)	0/41 (0·0)	0/39 (0·0)	0/37 (0·0)	0/10 (0·0)	0/7 (0·0)	0/9 (0·0)	0/6 (0·0)
Vomiting	0/21 (0·0)	0/25 (0·0)	0/21 (0·0)	0/19 (0·0)	0/37 (0·0)	0/41 (0·0)	0/39 (0·0)	0/37 (0·0)	0/10 (0·0)	0/7 (0·0)	0/9 (0·0)	0/6 (0·0)
Diarrhea	0/21 (0·0)	0/25 (0·0)	0/21 (0·0)	0/19 (0·0)	0/37 (0·0)	0/41 (0·0)	0/39 (0·0)	0/37 (0·0)	0/10 (0·0)	0/7 (0·0)	0/9 (0·0)	0/6 (0·0)
Itching	0/21 (0·0)	0/25 (0·0)	0/21 (0·0)	0/19 (0·0)	0/37 (0·0)	0/41 (0·0)	0/39 (0·0)	0/37 (0·0)	0/10 (0·0)	0/7 (0·0)	0/9 (0·0)	0/6 (0·0)

After treatment of 200 mg of albendazole, one participant (PSAC) reported a case of clinical malaria requiring inpatient hospitalization due to a serious adverse event. The participant received anti-malaria treatment and was released from the hospital within 24 h. No allergic reaction to any treatment arm was observed.

## 4 Discussion

The WHO has approved five different drugs and combinations against STH infections, of which albendazole is the most widely used [11]. Although albendazole has been licensed for human use since 1982, there have been limited studies of its efficacy against *T. trichiura* in PSAC and adults; and, the optimal dosages of the drug have not been determined in humans [3,10,17]. There are many studies on the single dose of 400 mg recommended by the WHO, which reveal albendazole has low efficacy against *T. trichiura* [6,9]. Despite the lack of effective alternative treatment against trichuriasis, to date only a handful of studies have been conducted on higher dosages and dose-finding studies in different age-groups have not been carried out [12,18,19]. As national control programs move towards elimination, treatment of all age groups becomes increasingly important to prevent spread of infection; therefore, optimal doses for PSAC, SAC, and adults are necessary [6].

This trial revealed that there is no remarkable dose response of albendazole against *T. trichiura* in PSAC, SAC, and adults within the observed range. The current recommended dose of 400 mg for SAC is not efficacious against *T. trichiura* and higher doses of 600 mg or 800 mg do not increase CRs or provide a greater reduction in infection intensity. These results are confirmed by the predictions of the *E*_max_ model, which show no visual difference between CRs between doses of albendazole for SAC. Furthermore, only the 400 mg dose of albendazole has an arithmetic ERR slightly surpassing the proposed ≥50% arithmetic ERR by the WHO [20]. Though recruitment was limited for PSAC and adults, a similar conclusion is plausible as CRs for albendazole were low in all treatment arms. Though this study was conducted in a single country only, we are convinced that the findings of this trial are generalizable to wider populations as possible confounding factors were limited (e.g., a rigorous diagnostic procedure was used). Moreover, strain differences resulting in varying albendazole susceptibility are not to be expected. In one of our recent trials, an albendazole combination with oxantel pamoate found similar CRs against STHs in both Laos and a nearby setting in Côte d'Ivoire [21].

It is impossible to conclude if 400 mg is the optimal dose for SAC and adults, since a 200 mg dose of albendazole was not tested. A flat dose response was not anticipated for any cohort, so the dose of 200 mg of albendazole was not included as a treatment arm when the trial was designed. In PSAC, however, a dose 200 mg of albendazole provided a similar efficacy and ERR as albendazole at 400 mg, hinting that the recommended higher dose does not provide any benefit in this age group for *T. trichiura* infection. However, this finding would need to be confirmed in a follow-up study as our recruitment targets for this age group were not met.

More females were recruited into the trial than males. Additional analysis stratified by sex showed a difference in CRs between females and males. Females and males had similar baseline infection intensities (Table 1) and it was confirmed through pharmacokinetic analyses that drug exposure of albendazole was similar across sex regardless of treatment arm (data not shown). Further studies will need to assess whether this difference may be due to chance or to evaluate the underlying reasons for this finding.

The findings of this trial are comparable to others on albendazole in the literature. In a recent network meta-analysis, Moser and colleagues found that 400 mg of albendazole had a limited efficacy against *T. trichiura* with CRs having decreased from 38·6% in 1999 to 16.4% in 2015 [10]. However, most of the trials included in the analysis limited their study populations to SAC [10]. In comparison, mebendazole was found to have a slightly higher CR (42·1%) with a single dose and higher CRs when given at 100 mg twice/day for 3 days (CR=63%) [10,19].

In 2000, Horton et al. summarized in a literature review that increasing the single dosage and using repeated doses improves the efficacy of albendazole against *T. trichiura* [12]. In contrast, in our study increased single dosages did not provide improved efficacy against *T. trichiura*. Differences could be attributed to decreasing efficacy over the last two decades, differing study design, or geographic area [12]. The scope of this trial did not include evaluation of repeated doses of albendazole, which shows mixed results in humans and would be more difficult to administer through control programs [19,22].

A low number of study participiants due to low prevalence in adults, and to a lesser extent, in PSAC, is a main limitation of our trial. Only 4·3% of adults screened were included in the trial as the majority (84·7%) of adults screened were found to be *T. trichiura* negative. The limited sample size of adults (*n* = 35 across four treatment arms) might have triggered higher CRs for the 400 mg and 600 mg treatment arms (55·6% and 50·0%, respectively). For PSAC, 62·1% of screened PSAC were negative for *T. trichiura* infection and only 22·5% of PSAC participated in the trial.

In conclusion, albendazole is not an effective treatment of *T. trichiura* even at the highest doses administered. Recently, a few trials have shown that combination therapy of albendazole and ivermectin is more efficacious against trichuriasis compared to monotherapy with the added benefit of *Strongyloides stercoralis* control [23]. This trial confirms to reduce the burden of STHs, new first-line treatments are needed, or combination therapy should be administered.

## Data sharing

Deidentified individual participant data reported in this research article and the study protocol are available upon request from the corresponding author after all findings are published. Data will be shared after the approval of a proposal by the authors for legitimate scientific purposes.

## Declaration of Competing Interest

We declare no competing interests.

## Appendix Supplementary materials

Image, application 1
