# Supplementary material for: Efficacy and safety of ascending dosages of albendazole against Trichuris trichiura in preschool-aged children, school-aged children and adults: A multi-cohort randomized controlled trial
Source: eClinicalMedicine. 2020 May 5;22:100335. doi: 10.1016/j.eclinm.2020.100335 (PMC7210508; doi:10.1016/j.eclinm.2020.100335)
Supplement: Supplementary file 1 [file mmc1.pdf]

Supplementary Material

Sample size calculations

Sample size determination for Patel et al. 'Efficacy and safety of ascending dosages of albendazole against Trichuris trichiura in preschool-aged children, school-aged children and adults: a multi-cohort randomized controlled trial'. The true apparent cure rates used in the simulations are shown in table 1.

Table 1: Assumed true apparent cure rates (in percent) used in the simulations

Doses [mg]	T.t. 1	T.t. 2	T.t. 3	T.t. 4	Hook 1	Hook 2	Hook 3	Hook 4
0	5	5	5	5	5	5	5	5
400	20	15	25	25	20	50	50	50
600	30	30	35	35	30	70	70	75
800	40	40	45	45	35	80	80	85

Simulation code

```
library(DoseFinding)
library(dplyr)
set.seed(11031103)

# True cure rates against Trichuris (S1-S3) and Hookworm (S4-S6)
Tcrs <- list(T1 = c(0.05, 0.20, 0.30, 0.40),
             T2 = c(0.05, 0.15, 0.30, 0.40),
             T3 = c(0.05, 0.25, 0.35, 0.45),
             T4 = c(0.05, 0.20, 0.30, 0.35),
             H1 = c(0.05, 0.50, 0.70, 0.80),
             H2 = c(0.05, 0.50, 0.75, 0.85),
             H3 = c(0.05, 0.40, 0.65, 0.75),
             H4 = c(0.05, 0.60, 0.75, 0.85))
Nseq <- seq(30, 50, 5)       # Samples sizes per arm to evaluate
lossFU <- 0.05               # 5% loss to follow-up
doses <- c(0, 400, 600, 800) # Doses
pred.range <- 0:800          # predict dose response curve from 0 to 800
loops <- 200                 # simulation runs per combination
counter <- 0
# data frame to store the simulation results
res <- data.frame(matrix(NA, loops * length(Nseq) * length(Tcrs), 5))
colnames(res) <- c("Seq","N","nfu","Median_0_800","Median_400_800")

for(k in 1:length(Tcrs)){
  Tcr <- Tcrs[[k]]
  for(j in Nseq){
    for(i in 1:loops){
      counter <- counter +  1
      res[counter, 1] <- k
      res[counter, 2] <- j
      n <- rbinom(4, j, 1-lossFU)
      res[counter, 3] <- sum(n)
```

```
      cured <- rbinom(4, n, Tcr)
      # ensure variation in placebo arm
      if(cured[1] == 0) cured[1] <- 1

      # fit logit model
      df <- data.frame(cbind(dose=doses, cured=cured, n=n))
      Crate <- df$cured/df$n
      fitBin <- glm(Crate ~ factor(doses) - 1, family = binomial, weights = df$n)
      drEst <- coef(fitBin)
      vCov <- vcov(fitBin)

      # fit dose range model
      gfit <- fitMod(doses, drEst, S=vCov, model = "emax", type = "general")
      logitPred <- predict(gfit, predType ="ls-means", doseSeq = pred.range, se.fit=TRUE)

      # confidence band on logit scale
      LB <- logitPred$fit-qnorm(0.975)*logitPred$se.fit
      UB <- logitPred$fit+qnorm(0.975)*logitPred$se.fit
      # confidence band on probability (cure rate) scale
      LB <- 1/(1+exp(-LB))
      UB <- 1/(1+exp(-UB))
      # Range 0 - 800 mg
      res[counter, 4] <- median(UB-LB)
      # Range 400 - 800 mg
      res[counter, 5] <- median(UB[401:801]-LB[401:801])
    }
  }
}

res$para <- ifelse(res$Seq <= 3, "Tri", "Hook")
sum.res <-res %>% group_by(para, N) %>%
  summarise(`median 0-800mg`=median(Median_0_800)*100/2,
            `median 400-800mg`=median(Median_400_800)*100/2,
            nFU =mean(nfu/4))
```

Table 2: One half length of the 95CI (percentage-pts)

para	N	median 0-800mg	median 400-800mg	nFU
Hook	30	12.9	11.6	28.5
Hook	35	11.9	10.7	33.3
Hook	40	11.0	10.0	38.0
Hook	45	10.5	9.4	42.7
Hook	50	9.9	8.9	47.5
Tri	30	12.3	12.1	28.5
Tri	35	11.3	11.2	33.2
Tri	40	10.5	10.4	38.0
Tri	45	9.9	9.9	42.7
Tri	50	9.3	9.4	47.5

Results Tables

Table S1: Cure rates and egg reduction rates against *Trichuris trichiura* of PSAC and adults at 3 weeks follow-up.

	PSAC				Adults			
	**ALB 200 mg**	**ALB 400 mg**	**ALB 600 mg**	**PLAC**	**ALB 400 mg**	**ALB 600 mg**	**ALB 800 mg**	**PLAC**
Positive before treatment	21	23	18	24	9	8	11	7
Cured after treatment	2	4	5	4	5	4	3	1
Observed CR [95% CI]	9·5 [1·2, 30·4]	17·4 [5·0, 38·8]	27·8 [9·7, 53·5]	16·7 [4·7, 37·4]	55·6 [21·2, 86·3]	50·0 [15·7, 84·3]	27·3 [6·0, 61·0]	14·3 [0·4, 57·9]
Predicted CR [95% CI]	17·2 [6·5, 38·3]	20·1 [10·3, 35·6]	22·5 [9·8, 43·8]	13·6 [5·1, 31·3]				
EPG geometric mean								
Baseline	273·3	327·4	324·7	285·2	257·2	223·4	291·4	198·6
3 weeks follow-up	99·1	42·4	37·4	59·6	6·0	11·8	21·6	29·4
Observed ERR [95% CI]	63·8 [33·1, 83·1]	87·1 [67·3, 95·3]	88·5 [70·4, 95·7]	79·1 [53·5, 91·2]	97·7 [86·7, 99·8]	94·7 [61·0, 99·6]	92·6 [71·9, 98·5]	85·2 [65·9, 95·3]
EPG arithmetic mean								
Baseline	491·4	914·1	1126·0	952·5	444·7	291·8	448·4	229·7
3 weeks follow-up	456·9	273·7	816·3	206·5	298·0	184·5	228·5	87·4
Observed ERR [95% CI]	7·0 [-25·3, 50·7]	70·1 [33·2, 86·1]	27·5 [-6·4, 76·5]	78·3 [32·6, 89·6]	33·0 [-18·9, 99·4]	36·8 [-105·0, 96·0]	49·0 [7·6, 94·4]	61·9 [36·1, 86·1]

Abbreviations: ALB, albendazole; CI, confidence interval; CR, cure rate; EPG, eggs per gram; ERR, egg reduction rate; PLAC, placebo; PSAC, preschool-aged children.

Table S2: Proportion of participants cured by treatment arm and sex in PSAC, SAC and adults.

		PSAC	SAC	Adults
**ALB 200 mg**	**F**	2/14 (14·3%)	na	na
	**M**	0/7 (0%)	na	na
**ALB 400 mg**	**F**	2/10 (20·0%)	3/14 (21·4%)	4/7 (57·1%)
	**M**	2/13 (15·4%)	4/29 (13·8%)	1/2 (50·0%)
**ALB 600 mg**	**F**	5/12 (41·7%)	5/18 (27·8%)	3/7 (42·9%)
	**M**	0/6 (0%)	6/25 (24·0%)	1/1 (100·0%)
**ALB 800 mg**	**F**	na	3/16 (18·8%)	1/7 (14·3%)
	**M**	na	4/25 (16·0%)	2/4 (50·0%)
**PLAC**	**F**	3/15 (20·0%)	1/25 (4·0%)	1/4 (25·0%)
	**M**	1/9 (11·1%)	2/21 (9·5%)	0/3 (0%)

Abbreviations: ALB, albendazole; F, female; M, male; na, not applicable; PLAC, placebo; PSAC, preschool-aged children; SAC, school-aged children.

Table S3: Cure rates and egg reduction rates against hookworm and *Ascaris lumbricoides* of participants at 3 weeks follow-up.

	PSAC				SAC				Adults			
	**ALB 200 mg**	**ALB 400 mg**	**ALB 600 mg**	**PLAC**	**ALB 400 mg**	**ALB 600 mg**	**ALB 800 mg**	**PLAC**	**ALB 400 mg**	**ALB 600 mg**	**ALB 800 mg**	**PLAC**
**Hookworm**												
Positive before treatment	0	1	0	0	1	2	3	1	1	0	0	1
Cured after treatment	na	1	na	na	0	2	3	0	1	na	na	0
CR	na	100	na	na	0·0	100	100	0·0	100	na	na	0·0
Ascaris lumbricoides												
Positive before treatment	6	3	1	2	18	9	13	13	2	0	3	0
Cured after treatment	5	2	1	0	16	9	11	5	2	na	3	na
CR	83·3	66·7	100	0	88·9	100	84·6	38·5	100	na	100	na
EPG geometric mean												
Baseline	3436·1	3958·5	30·0	4240·9	2781·0	3770·7	3262·2	1087·5	7261·8	na	1314·5	na
3 weeks follow-up	0·9	14·3	0	1711·4	0·9	0	0·9	190·8	0	na	0	na
ERR	99·9	99·6	100	59·7	99·9	100	99·9	82·5	100	na	100	na
EPG arithmetic mean												
Baseline	7091·0	4880·0	30·0	7989·0	12519·0	15535·3	8971·4	9067·7	8703·0	na	4612·0	na
3 weeks follow-up	7·0	1194·0	0	2436·0	125·0	0	51·2	7525·4	0	na	0	na
ERR	99·9	75·5	100	69·5	99·0	100	99·4	17·0	100	na	100	na

Abbreviations: ALB, albendazole; CR, cure rate; na, not applicable; PLAC, placebo; PSAC, preschool-aged children; SAC, school-aged children.

Protocol

Efficacy and safety of ascending dosages of albendazole against hookworm *and Trichuris trichiura* in preschool- and school-aged children and adults: a randomized controlled trial

**Protocol Number**	1		
**Version Number**	1.01	**Document Date**	22.01.2018
**Sponsor Contact**	Prof. Dr. Jennifer Keiser, Swiss Tropical and Public Health Institute, Tel.: +41 61 284-8218<br>Fax: +41 61 284-8105<br>E-mail: jennifer.keiser@unibas.ch		
**Principle Investigator**	Prof. Dr. Jennifer Keiser, Swiss Tropical and Public Health Institute, Tel.: +41 61 284-8218<br>Fax: +41 61 284-8105<br>E-mail: jennifer.keiser@unibas.ch		
**Funding Agency**	Bill and Melinda Gates Foundation		

1. General Information

I. List of investigators and other persons involved

Title	Names	Institution	Position	Function in trial
Prof. Dr.	Jennifer Keiser	Swiss Tropical and Public Health Institute (Swiss TPH)	Unit head	Principal Investigator
Dr.	Jean Coulibaly	Swiss TPH and Université Félix Hophouët-Boigny-Abidjan	Group leader	Co-PI
Dr.	Jessica Schulz	Swiss TPH	Postdoc	Co-investigator
Dr.	Yves N'Gbesso	Departement d'Agboville, Centre de Santé Urbain d'Azaguié, Azaguié	Medical doctor	Study physician
Dr.	Jan Hattendorf	Swiss TPH	Group leader	Statistician
MSc	Chandni Patel	Swiss TPH	PhD Student	Student

II. Signatures

Statistician

Signature		Date of Signature<br>22.01.2018
Name	Jan Hattendorf	
Title	Dr.	
Institution	Swiss Tropical and Public Health Institute	
Address	Department of Medical Parasitology and Infection Biology<br>Swiss Tropical and Public Health Institute, Socinstr. 57<br>CH- 4002 Basel, Switzerland	
Phone	+41 61 284-8193	

Principal investigator

Signature		Date of Signature<br>22.01.2018
Name	Jennifer Keiser	
Title	Prof.	
Institution	Swiss Tropical and Public Health Institute	
Address	Department of Medical Parasitology and Infection Biology<br>Swiss Tropical and Public Health Institute, Socinstr. 57<br>CH- 4002 Basel, Switzerland	
Phone	+41 61 284-8218	

I have read this protocol and agree that it contains all necessary details for carrying out this trial. I will conduct the trial as outlined herein and will complete the trial within the time designated.
I will provide copies of the protocol and all pertinent information to all individuals responsible to me who assist in the conduct of this trial. I will discuss this material with them to ensure they are fully informed regarding the drug and the conduct of the trial.
I will use only the informed consent forms approved by the Sponsor or its representative and will fulfill all responsibilities for submitting pertinent information to the Independent Ethics Committees responsible for this trial.
I agree that the Sponsor or its representatives shall have access to any source documents from which Case Report Form information may have been generated.

Co-Principal investigator

Signature		Date of Signature<br>22.01.2018
Name	Jean Coulibaly	
Title	Dr	
Institution	Swiss Tropical and Public Health Institute/Université Félix Houphouët-Boigny	
Address	23 BP 915 Abidjan 23	
Phone	+225 0500 8223/+225 0209 1401	

Co- investigator

Signature		Date of Signature<br>22.01.2018
Name	Jessica Schulz	
Title	Dr.	
Institution	Swiss Tropical and Public Health Institute	
Address	Department of Medical Parasitology and Infection Biology<br>Swiss Tropical and Public Health Institute, Socinstr. 57<br>CH- 4002 Basel, Switzerland	
Phone	+41 61 207 15 06	

III. Table of contents

1. General Information 2
2. Background information 12
3. Trial objective and purpose 14
4. Methodology 14
4.1. Primary and secondary endpoint 14
4.2. Type of trial 15
4.3. Trial design 15
4.3.1. Baseline survey 15
4.3.2. Assessment of efficacy after treatment 17
4.3.3. Pharmacokinetic studies 17
4.4. Measure to minimize bias 18
4.5. Study duration and duration of subject participation 19
4.6. Schedule of visits 19
5. Selection of the trial subjects 19
5.1. Recruitment 19
5.2. Inclusion criteria 20
5.3. Exclusion criteria 20
5.4. Criteria for discontinuation of trial 21
5.5. Treatment of subjects 21
5.6. Concomitant therapy 22
6. Assessment of safety 22
6.1. Adverse event definitions 23
6.1.1. Severity grading 23
6.1.2. Relatedness 24
6.1.3. Expectedness 24
6.1.4. Serious adverse events 24
6.1.5. Suspected unexpected serious adverse reactions 25
6.2. Methods of recording and assessing adverse events 25

6.3. Reporting of serious adverse events 26
6.4. Safety reporting to Health Authorities and Ethics Committees 27
7. Statistics 27
7.1. Definition of primary endpoint 27
7.2. Justification of number of trial subject 27
7.3. Description of statistical methods 28
8. Data management and data quality control 29
8.1. Data collection 29
8.2. Ethical, legal and security issues 30
8.3. Data storage and preservation 30
9. Duties of the investigator 30
9.1. Investigator's confirmation 30
9.2. Damage coverage 31
9.3. Project management 31
10. Ethical considerations 31
10.1. Independent Ethics Committee (IEC) 31
10.2. Evaluation of the risk-benefit ratio 32
10.3. Subject information and consent 32
10.4. Subject confidentiality 33
10.5. Subjects requiring particular protection 33
10.6. Other aspects 33
11. Quality control and quality assurance 33
11.1. Monitoring and auditing 33
11.2. Access to data, handling of data and samples (data protection), archiving (place, duration) and destruction 34
11.3. Data entered directly in the Case Report Form (CRF) – definition of source data 34
11.4. Data and safety monitoring board (WHO) / data monitoring committee (EU/FDA)....35
11.5. Study Documents: Translations - Reference language 35
12. Dissemination of results and publication 35
13. References 36

IV. Abbreviations

AE	Adverse event
AUC	Area under the curve
CI	Confidence interval
CR	Cure rate
CRF	Case report form
DALYs	Disability-adjusted life years
DBS	Dried blood spot
DSMB	Data and safety monitoring board
EKNZ	Ethikkomission Nordwest- und Zentralschweiz
EPG	Eggs per gram
ERR	Egg reduction rate
GCP	Good clinical practice
Hb	Hemoglobin
ICH	International council for harmonisation of technical requirements for pharmaceuticals for human use
IEC	Independent ethics committee
LC-MS/MS	Liquid chromatography tandem mass spectrometry
MIC	Minimal inhibitory concentration
NLME	Nonlinear mixed-effects
PI	Principal investigator
PK	Pharmacokinetic
PKPD	Pharmacometric-pharmacodynamic
SAE	Serious adverse event
STH	Soil-transmitted helminth
WHO	World Health Organization

V. Synopsis

**Study Title**	Efficacy and safety of ascending dosages of albendazole against *T. trichiura* and hookworm in preschool- and school-aged children and adults: a randomized controlled trial
**Short title**	Albendazole dose finding and pharmacokinetics in children and adults
**Study Type**	Phase 2 trial
**Sample size**	1040
**Indication**	*T. trichiura* and hookworm infection (eggs in stool)
**Investigational Product and Reference Treatment**	Albendazole
**Protocol Number, Date and Version**	1, 25.02.2018, v1.01
**Trial registration**	ClinicalTrials.gov: NCT03527745
**Study Rationale**	To determine the range of doses of albendazole against infection with *T. trichiura* and hookworm which are effective and tolerated by preschool-aged children, school-aged children and adults.
**Study Objectives**	To determine the efficacy and safety of ascending oral albendazole dosages in children and adults infected with *T. trichiura* and in children and adults infected with hookworm. In preschool-aged children: (i) 200 mg, (ii) 400 mg and (iii) 600 mg; and in schoolchildren and adults: (i) 200 mg (only for hookworm infections), (ii) 400 mg, (iii) 600 mg and (iv) 800 mg will be compared against both parasites (*T. trichiura* and hookworm). Additionally, albendazole disposition will be measured in children and adults using a microsampling device.<br><br>Our **primary objective** is to determine the dose-response based on cure rates of albendazole in (i) preschool-aged children (2-5 years), (ii) school-aged children (6-12 years) and (iii) adults (≥ 21 years) infected with *T. trichiura* and in (i) preschool-aged children (2-5 years), (ii) school-aged children (6-12 years) and (iii) adults (≥ 21 years) infected with hookworm.<br><br>The **secondary objectives** of the trial are:<br><br>a) To determine the efficacy based on egg reduction

	rates of albendazole in preschool-aged children, school-aged children and adults;<br>b) To determine an exposure (including Cmax, AUC and tmax)-response correlation of albendazole in preschool-aged (2-5 years), school-aged children (6-12 years), and adults (≥ 21 years);<br>c) To evaluate the safety and tolerability of albendazole in preschool-aged children, school-aged children and adults; and<br>d) To determine the efficacy against concomitant soil-transmitted helminthiasis (*Ascaris lumbricoides*)<br>e) To assess genetic heterogeneity in hookworm and *T. trichiura* populations with regard to the efficacy of different doses of albendazole.
**Study design**	Single blind, randomized controlled trial (participants and lab technicians are blinded)
**Study product / intervention**	Administration of a single oral dose of albendazole
**Comparator(s)**	Matching placebo (purchased from Fagron)
**Key inclusion / Exclusion criteria**	Inclusion: preschool-aged children (2-5 years), school-aged children (6-12 years) and adults (≥21 years) infected with *T. trichiura* or hookworm, absence of major systemic illnesses, written informed consent signed by parents and/or caregivers for minors; and oral assent by school-aged children.<br>Exclusion: Any abnormal medical conditions or chronic disease, negative diagnostic result for *T. trichiura* and hookworm, pregnancy, no written informed consent.
**Primary Endpoints**	CR on *T. trichiura* and hookworm
**Secondary Endpoints**	ERR against *T. trichiura* and hookworm and safety, pharmacokinetic parameters, genetic heterogeneity in hookworm and *T. trichiura* populations
**Exploratory Endpoints**	None
**Interim Analyses**	None
**Study Duration**	6 months
**Schedule**	08/2018 of first-participant in (planned)<br>04/2019 of last-participant out (planned)
**Measurements & procedures**	At baseline, two stool samples will be collected, if possible, on two consecutive days or otherwise within a maximum of 5 days. The medical history of the participants will be assessed with a

	standardized questionnaire (for preschool-aged children this will be administered to the caregiver of the child). In addition a clinical examination will be performed by the study physician and venipuncture to examine biochemical parameters in the blood will be carried out by study nurses before treatment.<br>Adults, children and caregivers of preschool-aged children will also be interviewed before treatment, 3 and 24 hours after treatment, and at follow-up (2-3 weeks after treatment) about the occurrence of adverse events. The efficacy of the treatment will be determined 14–21 days post-treatment by collecting another two stool samples. All stool samples will be examined with duplicate Kato-Katz thick smears. A subsample of children and adults will be sampled using finger pricking for micro-blood sampling at 0, 1, 2, 3, 4, 6, 8, 24 hours post-dosing to evaluate pharmacokinetic parameters.
**Statistical Analyses**	An available case analysis (full analysis set according to the intention to treat principle) will be performed, including all subjects with primary endpoint data. Supplementary, a per-protocol analysis will be conducted. CRs will be calculated as the percentage of egg-positive subjects at baseline who become egg-negative after treatment.<br>Geometric mean egg counts will be calculated for the different treatment arms before and after treatment to assess the corresponding ERRs Bootstrap resampling method with 5,000 replicates will be used to calculate 95% confidence intervals (CIs) for ERRs. E_{max} models using the DoseFinding package of the statistical software environment R will be implemented to predict the dose-response curves in terms of CRs and ERRs.<br>Noncompartmental and nonlinear mixed-effects (NLME) modeling will be used to determine PK parameters.
**GCP statement**	This study will be conducted in compliance with the protocol, the current version of the Declaration of Helsinki, ICH-GCP E6 (R2) guidelines as well as all national legal and regulatory requirements.
**Key explanation for the inclusion of children**	This study will be carried out in children, since an infection with *T. trichiura* and hookworm occurs most often in children and they are the main targets of preventive chemotherapy programs. Moreover, the exact dosage of albendazole in preschool and school-aged children has not yet been determined.
**Recruitment procedure**	Participants will be recruited from rural communities situated in Côte d'Ivoire and identified to have at least a moderate infection risk (prevalence: 10-49%) of *T. trichiura* or hookworm.

**Coverage of damages**	Winterthur Police Nr. 4746321, BERACA Côte d'Ivoire, No: to be issued
**Storage of data and samples for future research aims**	After the study has been completed all samples will be destroyed and case report forms will be kept for a minimum of 15 years (chapter 10).
**Conflict of interest in relation to the investigated drugs**	We declare no conflict of interest in relation to the investigated drugs.

2. Background information

Parasitic worm diseases such as infection with soil-transmitted helminths (STHs) that include infection with *Ascaris lumbricoides*, *Trichuris trichiura*, and hookworm still affect hundreds of millions of people mostly in the tropics and subtropics [1]. In terms of morbidity, they contribute to an estimated 3.3 million disability-adjusted life years (DALYs) lost globally according to recent estimates [2]. The symptoms of STH infections are non-specific and may only be apparent in heavily infected individuals. Infection is typically most intense and debilitating in children. Chronically infected children might suffer from malnutrition, physical and cognitive retardation, and reduced work performance in older age [3].

For disease control, preventive chemotherapy represents the cornerstone and is advocated by the World Health Organization (WHO) as a strategy with a rapid impact on morbidity control and easily implementable at a large scale by trained personnel outside the health sector (e.g. teachers) [4]. Currently there are five drugs on the WHO model list of essential drugs against STH infections and these drugs have been widely and effectively used [4, 5]. Among these five, albendazole and mebendazole, which belong to the benzimidazoles, are considered as treatment of choice against *A. lumbricoides*, *T. trichiura* and hookworm infections [4]. However, both drugs show only limited efficacy against *T. trichiura* infection (cure rates of 30% and 42%, respectively) [5] and only albendazole is moderately efficacious against hookworm when used as single oral doses.

Surprisingly, little is known on albendazole. The optimal dosages, as for many other anthelminthics, were never determined for albendazole and the 400 mg dose widely used is rather empirical. Unpublished data suggest higher efficacy when albendazole is used at higher dosages (John Horton, unpublished observation). Different dosages and regimen (single and multiple dose) of albendazole were tested against soil-transmitted helminthiasis but a thorough dose-response relationship study testing different doses in the same trial were not conducted to date [6]. A single dose of 800 mg albendazole was the highest single dose tested to date against soil-transmitted helminthiasis in school-aged children and adults [6] and other diseases e.g. *Giardia* infections [7], which represents a well-tolerated dose; therefore, was selected as the highest dose in our study for school-aged children and adults. Preschool-aged children will be treated with a slightly lower dose of 600 mg albendazole (John Horton personal communication), which will not pose any health risk as albendazole is an exceptionally safe drug. 400 mg is the standard dose for preschool-aged children, school-aged children and adults recommended by WHO [5]. To be able to determine the dose response curve, 200 mg was selected as the lowest dose in the different cohorts. In addition, pharmacokinetic studies have not yet been conducted with albendazole in individuals infected with hookworm or *T. trichiura*; it is not known whether a pharmacokinetic

(PK)/pharmacodynamic relationship exists and whether there are differences in the behavior between different age groups.

In view of remaining knowledge gaps in exact doses of albendazole in different population strata, our study aims at thoroughly investigating the safety and efficacy of ascending doses of albendazole (preschool-aged childreners: 200, 400 and 600 mg; school-aged children and adults: 200, 400, 600 and 800 mg) against *T. trichiura* and hookworm infections complemented by PK studies.

3. Trial objective and purpose

The primary study objective is:
To determine the dose-response of oral albendazole dosages: i) 200 mg, ii) 400 mg and iii) 600 mg versus iv) placebo in preschool-aged children and i) 400 mg, ii) 600 mg and iii) 800 mg versus v) placebo in school-aged children and adults infected with *T. trichiura* and i) 200 mg, ii) 400 mg and iii) 600 mg versus iv) placebo in preschool-aged children and i) 200 mg ii) 400 mg, iii) 600 mg and iv) 800 mg versus v) placebo in school-aged children and adults infected with hookworm.

The secondary study objectives are:

1.) To determine the efficacy based on egg reduction rates of albendazole in preschool-aged children, school-aged children and adults;

2.) To measure albendazole disposition using a microsampling technology (DBS or Mitra®) to determine an exposure (including Cmax, AUC and tmax)-response correlation of albendazole in preschool-aged (2-5 years), school-aged children (6-12 years) and adults (≥ 21 years);

3.) To evaluate the safety and tolerability of albendazole in preschool-aged children, school-aged children and adults;

4.) To determine the efficacy against concomitant soil-transmitted helminthiasis (*Ascaris lumbricoides*); and

5.) To assess genetic heterogeneity in hookworm and *T. trichiura* populations with regard to the efficacy of different doses of albendazole.

4. Methodology

4.1. Primary and secondary endpoint

Cure rate (CR) (primary endpoint, i.e. conversion from being egg positive pre-treatment to egg negative post-treatment) and egg reduction rate (ERR) (secondary endpoint). In addition, key pharmacokinetic parameters will be determined and safety of treatment evaluated (secondary endpoints).

4.2. Type of trial

Single blind, randomized controlled trial

4.3. Trial design

4.3.1. Baseline survey

Stool samples collection

Two stool samples will be collected from each participant until a total of 560 hookworm-infected participants (160 preschool-aged children, 200 school-aged children and 200 adults) and 480 *T. trichiura* infected participants (160 preschool-aged children, 160 school-aged children and 160 adults) are identified.

The Kato-Katz technique will be used for the quantitative assessment of STH infections. Two stool samples will be collected from each participant before treatment and from each sample duplicate Kato-Katz thick smears (41.7 mg each) will be prepared [8]. The slides will be analyzed under a microscope by experienced technicians and a subsequent independent quality control of sample results (approximately 10%) will be conducted. Results are considered correct if the following tolerance margin is not exceeded: (i) No difference in presence/absence of hookworm, *A. lumbricoides* and *T. trichiura*, (ii) egg counts are +/-10 eggs for counts ≤100 eggs or +/-20% for counts >100 eggs (for each species separately) [9]. In case discrepancies above the tolerance margin are noted in one or more slides, all slides are re-read by the local technicians. The new results are discussed, so that in case of discordant results, slides can be re-evaluated to reach consensus. Infection intensity expressed as the arithmetic and geometric mean egg counts per gram of stool (EPG) will be calculated for each treatment arm. All microscopically analyzed quadruplicate Kato-Katz thick smears will be destroyed within one day (after passing the quality control). Since drug efficacy could be influenced by genetic aspects, ~2 g of stool sample from each participant will be preserved in ethanol for further genetic analyses of hookworm and *T. trichiura* in Côte d'Ivoire and Switzerland.

Clinical examination and biological exams

At baseline, the medical history of *T. trichiura* and hookworm-infected participants in the study will be assessed with a clinical and physical examination carried out by the study clinician, who will assess weight, height, body temperature, pulse rate and blood pressure. Rapid diagnostic tests for malaria will be done to assess the presence of a *Plasmodium* spp. co-infection. Hemoglobin (Hb) levels will be detected using HemoCue to exclude severely anemic participants (below 80 g/l and 70 g/l Hb in school-aged and preschool-aged children,

respectively, according to WHO). Female participants above 10 years will be asked to produce a urine sample for pregnancy testing. Vital organ (kidney, liver etc.) functions will be evaluated before treatment and between 14 and 21 days after treatment through a biochemical assessment of 4 ml of venous blood. We will assess urea, creatinine, bilirubin, azotemia, Alanine Amino Transferase (ALAT), Aspartate Amino Transferase (ASAT) and blood cell counts (hematocrit, erythrocyte, platelets).

The six study cohorts (adults and school-aged children are summarized in one flow chart because the treatment schedule is identical) are presented in the figure below:

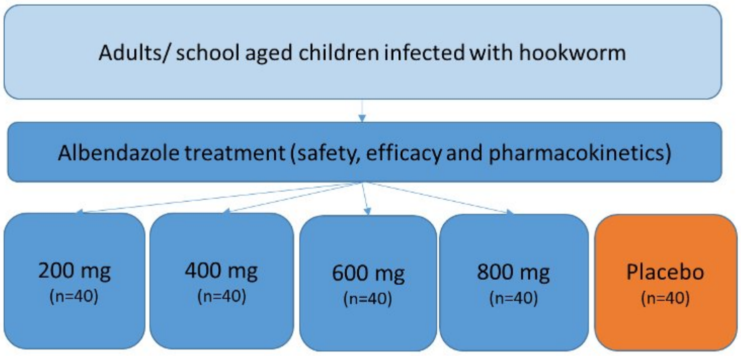

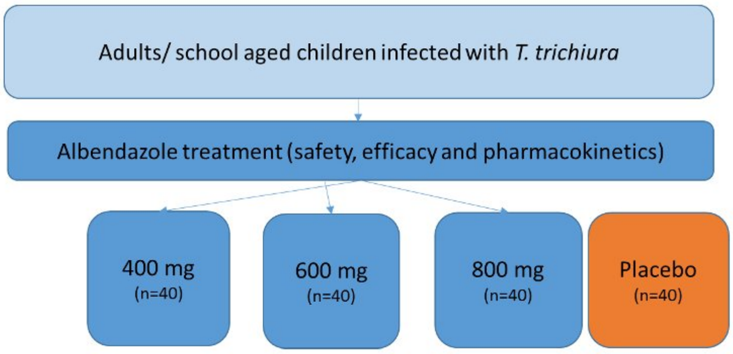

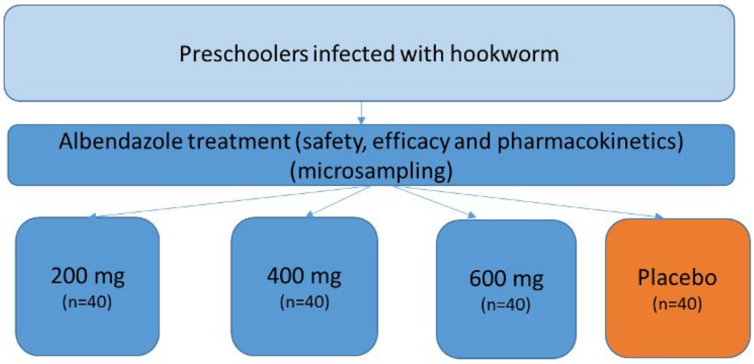

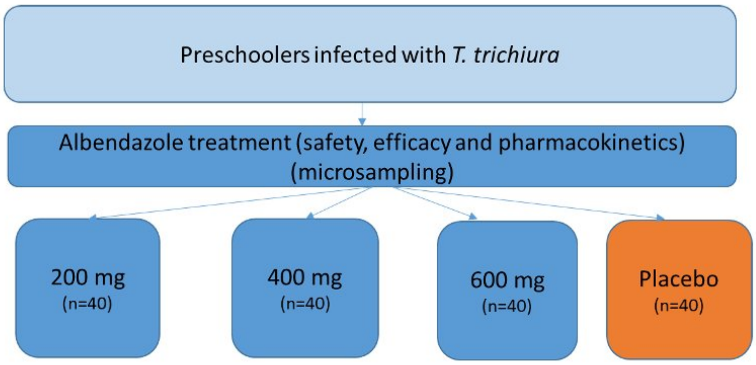

4.3.2. Assessment of efficacy after treatment

The efficacy of the treatment will be determined 14-21 days post-treatment by collecting another two stool samples which will be microscopically examined for *T. trichiura* and hookworm and for co-infections (*A. lumbricoides*) using duplicate Kato-Katz thick smears.

Participants will be considered *T. trichiura* and hookworm cured if no eggs have been found in the stool. EPG will be assessed by adding up the egg counts from the quadruplicate Kato-Katz thick smears and multiplying this number by a factor of six. Geometric and arithmetic mean egg counts will be calculated for the different treatment arms before and after treatment to assess the corresponding ERRs.

At the end of the study, all participants remaining positive for *T. trichiura* or hookworm will be treated with 400 mg albendazole, the current standard treatment.

4.3.3. Pharmacokinetic studies

Pharmacokinetic studies will be performed in a maximum of 20 participants per treatment arm (520 patients in total). Upon oral intake, albendazole gets metabolized first to sulfoxide and further to sulfone, whereas the sulfoxide is thought to be the most active compound to treat STH infection. In order to evaluate potential differences of PK parameters, including the metabolization rate in different populations, all three compounds, namely albendazole, albendazole sulfoxide and albendazole sulfone, will be quantified with time. Two blood micro-sampling techniques, namely dried blood spot (DBS) and Mitra® are currently under investigation to quantify albendazole and its metabolites in human blood samples. Consequently, whole blood, plasma, DBS and Mitra® blood samples were collected in a previous clinical trial in rural Côte d'Ivoire with 10 adult volunteers who received the standard dose of albendazole. Methods to extract and quantify albendazole and its metabolites in all four matrixes are currently validated in order to demonstrate that the capillary and venous blood results correlate satisfactoryly. Once validation of the analytical method has been completed, the best blood micro-sampling device, DBS or Mitra®, will be chosen as a tool for these albendazole population pharmacokinetic studies in preschool-aged children, school-aged children and adults.

In more detail, capillary blood (± 0.1 ml) will be collected by middle or ring finger tip puncture using a finger pricker (e.g. Accu-chek Softclix Pro®, Roche). Sampling will be conducted at 0, 1, 2, 3, 4, 6, 8, 24 hours post-dosing. The time points might be slightly adapted (shifted or reduced) in case the purpose for optimization is seen after the analyses of the samples of the 10 adult patients has been completed. A few drops of blood will be transferred at each time point on DBS filter paper (Whatman) or Mitra® microsampling device and dried for approximately 1 hour. The DBS cards or Mitra® will be transported to Swiss TPH in Basel and stored at -80° C until analysis. Albendazole and its metabolites will be quantified using a validated liquid chromatography tandem mass spectrometry (LC-MS/MS) method. Drug concentrations will be calculated by interpolation from a calibration curve with a foreseen limit of quantification of approximately 1-5 ng/ml. Quality control samples will be included in the study and its measured concentrations used to determine between-run and overall precision and accuracy of the analysis.

4.4. Measure to minimize bias

Study participants eligible for treatment will be randomly assigned to one of the treatment arms using a computer-generated, stratified block randomization code. The random allocation sequence with varying random blocks of 4 and 8 (or 5 and 10, respectively) stratified by 2 levels of baseline infection intensity (light and moderate *T. trichiura* and hookworm infections) will be provided by a statistician. Study-site investigators will be aware

of the study group assignment, whereas participants and laboratory technicians will be blinded.

Placebo controlled trials reduce the risk of potential bias that can also interfere with the observed outcomes. In previous studies (e.g. Coulibaly et al. 2017 Lancet Global Health), we have observed moderate cure and egg reduction rates in placebo treated children likely due to the low sensitivity of anthelminthic diagnostics. Children treated with placebo will be treated at follow up (3-weeks post treatment) with 400 mg albendazole. This treatment delay does not cause any harm.

4.5. Study duration and duration of subject participation

The trial will last for a maximum of 6 months. The screening for the baseline will start four to six weeks prior to the treatment. Follow up screening will take place 14-21 days post-treatment and last for about three weeks. Schedules of visits are summarized below.

4.6. Schedule of visits

	Screening					Follow up
	-28 to -1 days	Hours				2-3 weeks
		0	Randomization and treatment	1-8	24	
**Informed consent**	X					
**Diagnosis (stool examination)**	X					X
**Demographics**	X					
**Medical history**		X				
**Clinical examination**		X				
**Biochemical parameter**		X				X
**PK sampling**		X		X	X	
**Capturing AEs**				X (3h)	X	X
**Capturing SAE**				X (3h)	X	X

5. Selection of the trial subjects

5.1. Recruitment

The study will be carried out in different age groups in areas endemic for *T. trichiura* and hookworm in Côte d'Ivoire. Age groups investigated include preschool-aged children 2-5 years old, school-aged children 6-12 years old and adults of 21 years or more.

The parents/guardians of the children and potential participating adults will be invited to participate in an informational meeting. Community members will be encouraged to ask questions in an open discussion forum. The parents/guardians will then be approached individually to explain and discuss the purpose and procedures of the study, including potential benefits and risks.

5.2. Inclusion criteria

1. Male and female preschool-aged children (2-5 years), school-aged children (6-12 years) and adults (≥21 years) infected with *T. trichiura*/hookworm.
2. Written informed consent signed by study participant/parent/guardian and written assent in the case of school-aged children.
3. Positive for *T. trichiura*/hookworm by at least two slides of the quadruple Kato-Katz thick smears and infection intensities of at least 100 eggs per gram of stool (EPG).
4. Agree to comply with study procedures, including provision of two stool samples at the beginning (baseline) and approximately three weeks after treatment (follow-up).

5.3. Exclusion criteria

1. Presence of acute or uncontrolled systemic illnesses (e.g. severe anemia, infection, clinical malaria) as assessed by a medical doctor, upon initial clinical assessment and liver function tests.
2. Known or reported history of chronic illness such as HIV, acute or chronic hepatitis, cancer, diabetes, chronic heart disease or renal disease.
3. Prior treatment with anthelmintics (eg, diethylcarbamazine [DEC], suramin, ivermectin, mebendazole or albendazole) within 4 weeks before planned test article administration.
4. Received any investigational drugs or investigational devices within 4 weeks before administration of test article that may confound safety and/or efficacy assessments.
5. Known or suspected allergy to benzimidazoles.
6. Pregnant (urine testing) or breastfeeding women.

Participants who were diagnosed with a STH infection, but who were excluded from the study due to one or several of the above-mentioned exclusion criteria, including withdrawals will be offered standard anthelminthic treatment (albendazole). Children suffering from

clinical malaria (fever plus positive diagnostic test) will be treated according to national guidelines but will not be included in the trial.

5.4. Criteria for discontinuation of trial

A subject can be discontinued from the study for the following reasons:

1. Withdraws from the study (this can happen anytime as participation is voluntary and there are no further obligations once a child withdraws).
2. At the discretion of the Principal Investigator, if the participant is not compliant to the requirements of the protocol or participation places the study subject at undue risk.

Discontinued subjects will not be replaced. If, for any reason, a subject is discontinued from the study before the end of treatment evaluations, the safety procedures planned (adverse events monitoring) will be conducted.

5.5. Treatment of subjects

400 mg chewable tablets of albendazole (Zentel®) will be obtained from Glaxo Smith Kline. Tablets will be split to obtain 200 mg and 600 mg dosages. Matching placebo tablets will be obtained from Fagron, Germany.

Participants will receive a small breakfast two hours prior to treatment and breakfast and lunch during the day once absorption of the drug is complete.

All drugs will be administered in the presence of the investigator(s), and ingestion confirmed. This will be recorded with the time and date of dosing. Subjects will be asked not to take any drugs other than those prescribed by the study medical team. After ingestion of the medication, the subjects will be observed for 3 hours to ensure retention of the drug. Vomiting within 1-hour post-dosing will require re-dosing. The subjects will not be allowed more than one repeated dose. No re-administration will be needed for subjects vomiting after one hour.

On each treatment day about 40 participants will be treated with the different dosages. In case any serious adverse events will be observed, the study will be put on hold until the data has been reviewed and a decision taken within the entire study team whether the study can be continued or has to be stopped. On the treatment day, participants presenting a serious medical situation as judged by the physician of the project will be kept at the hospital level under close supervision by the medical team, after the parents (for children) have been informed on the reasons for the prolonged hospitalization. Also, in order not to break the surveillance of the undesirable effects of those whom we will allow to go back home at the end of the treatment day, security measures will be taken: (i) a card containing at least three phone numbers of the investigators and the study physician will be given to the parents

(children) or the participant himself (adults). This card including the phone numbers on it will remain valid through the duration of the clinical trial; (ii) for the 24-hour period following treatment, a telephone unit transfer of at least 2000 CFA will be transferred to each participant or the participant's parent so that they can call the investigators at any time; (iii) a medical team with an emergency kit will stay within the community for 24 hours following the treatment; and (iv) on the basis of an agreement to be signed with the responsible health district, an ambulance will be made available to enable rapid evacuation if necessary. All the financial implications of these arrangements will be fully supported by the project.
The Principal Investigator is responsible for drug accountability at the study site. Maintaining drug accountability includes careful and systematic study drug storage, handling, dispensing and documentation of administration.
We will be working with children or adults in rural areas. Participants who are involved in agricultural activities and migrating between locations will be difficult to follow by the investigators. We will provide them with phone numbers and follow them up with the help of health workers in case adverse events occur.

5.6. Concomitant therapy

All medications taken one month before and during the study period must until the last stool examination between day 14 and 21 (follow-up) be recorded with indication, dose regimen, date and time of administration.
Medication(s)/treatment(s) permitted during the trial

- Analgesics and antipyretics are allowed to be given to the subjects in case of fever, antiemetics to prevent nausea and vomiting and/or antibiotics to prevent or treat bacterial superinfection.

Medication(s)/treatment(s) NOT permitted during the trial

- No other active drugs or use of grapefruit juice against helminths are permitted during the trial

6. Assessment of safety

Few adverse events have been reported following albendazole administration in STH-infected individuals. The most common adverse events were abdominal cramps, headache, nausea, diarrhea, fever and vertigo [10]. The safety profile of albendazole will be assessed through the recording, reporting and analysis of baseline medical conditions, adverse events and a physical and clinical examination as described in section 4.3.1.

6.1. Adverse event definitions

The term "adverse event" could include any of the following events which develop or increase in severity during the course of the study, after administration of the study product:

a) Any unfavourable and unintended signs, symptoms or disease temporally associated with the use of a medicinal product, whether or not considered related to the condition under study and the study product;
b) Any abnormality detected during physical and clinical examination and whole blood analysis.

The medical conditions present at the initial trial visit that do not worsen in severity or frequency during the trial will not be defined as adverse events but be considered baseline medical conditions.

The observation time for adverse events starts when the treatment is initiated until the last follow-up examination (21 days after drug administration).
These data will be recorded on the appropriate CRF sections, regardless of whether they are thought to be associated with the study or the drug under investigation. Associated with the use of the drug means that there is a reasonable possibility that the event may have been caused by the drug (see also relatedness definitions below).

6.1.1. Severity grading

Adverse signs or symptoms will be graded by the Investigator as mild, moderate, severe or life threatening according to the following definitions (*https://evs.nci.nih.gov/ftp1/CTCAE/CTCAE_4.03_2010-06-14_QuickReference_5x7.pdf*):

Grade	Definition
1	Mild: the subject is aware of the event or symptom, but the event or symptom is easily tolerated.
2	Moderate: the subject experiences sufficient discomfort to interfere with or reduce his or her usual level of activity.
3	Severe: significant impairment of functioning: the subject is unable to carry out his or her usual activities.

4	Life threatening or disabling
5	Death related to adverse events

6.1.2. Relatedness

Relatedness will be assessed as defined below based on the temporal relationship between the adverse event and the treatment, known side effects of treatment, medical history, concomitant medication, course of the underlying disease and trial procedures.

Possibly related: an adverse event which can medically (pharmacologically/clinically) be attributed to the study treatment.
Unrelated: an adverse event which is not reasonably related to the study treatment. A reasonable alternative explanation must be available.

6.1.3. Expectedness

Expected adverse event: Any adverse event of albendazole reported in the literature or on the drug package leaflet and listed in the consent form. These adverse events are detailed in the drug package leaflet (Appendix 1).
Unexpected adverse event: Any adverse event, the nature, frequency, specificity or severity of which is unanticipated and not consistent with the available risk information described for these drugs.

6.1.4. Serious adverse events

According to the ICH "Clinical Safety Data Management: Definitions and standards for expedited Reporting E2A" (http://www.ich.org/fileadmin/Public_Web_Site/ICH_Products/Guidelines/Efficacy/E2A/Step4/E2A_Guideline.pdf), a serious adverse event (SAE) includes any event that:

1. results in death;
2. is life threatening, meaning, the subject was, in the view of the Investigator, at immediate risk of death from the reaction as it occurred, *i.e.*, it does not include a reaction that, had it occurred in a more serious form, might have caused death;
3. results in persistent or significant disability/incapacity, *i.e.*, the event causes a substantial disruption of a person's ability to conduct normal life functions;
4. requires, in-patient hospitalization or prolongation of existing hospitalization;

5. creates a congenital anomaly or birth defect (not relevant for this study);
6. is an important medical event, based upon appropriate medical judgment, that may jeopardize the patient or subject or may require medical or surgical intervention to prevent one of the other outcomes defining serious.

A “severe” adverse event does not necessarily meet the criteria for a “serious” adverse event. Serious adverse events are reported from consent to the last follow-up examination (14-21 days post-treatment).

Serious adverse events that are still ongoing at the end of the study period will be followed up to determine the final outcome.

The causality of any serious adverse event that occurs after the study period and its possible relatedness to the study treatment or study participation will also be assessed by the investigators as described in section 6.1.2.

6.1.5. Suspected unexpected serious adverse reactions

A suspected unexpected serious adverse reaction (SUSAR) is an unexpected adverse reaction which also meets the definition of serious adverse events.

6.2. Methods of recording and assessing adverse events

Subjects will be kept for observation for at least 3 hours following treatment for any acute adverse events. If there is any abnormal finding, the local study physician will perform a full clinical and physical examination and findings will be recorded. An emergency kit will be available on site to treat any medical conditions that warrant urgent medical intervention. Adults, school-aged children and the mother of preschool-aged children will also be interviewed 3 and 24 hours after treatment and at follow-up (2-3 weeks after treatment) about the occurrence of adverse events (see chapter 4.6).
Information on all adverse events (onset, duration, intensity, seriousness and causality) will be immediately entered in the appropriate adverse event module of the case report form (CRF) that serves as source document. For all adverse events, sufficient information will be pursued and/or obtained so as to permit i) an adequate determination of the seriousness of the event (i.e. whether the event should be classified as a serious adverse event); ii) an assessment of the casual relationship between the adverse event and the study treatments and iii) an assessment of intensity of adverse events will be judged by the study physician.

All serious adverse events or SUSARs must be reported as described in Section 6.3.

6.3. Reporting of serious adverse events

Any study-related unanticipated problem posing risk of harm to subjects or others, and any type of serious adverse event, will be immediately (within a maximum of 24 hours after becoming aware of the event) notified to the principal study sponsor-investigator and co-principal investigator:

Prof. Dr. Jennifer Keiser (Sponsor-investigator)
Swiss Tropical and Public Health Institute
Socinstrasse 57, 4051 Basel, Switzerland
Tel.: +41 61 284-8218
Fax: +41 61 284-8105
E-mail: jennifer.keiser@swisstph.ch

Dr. Jean T. Coulibaly (Co-principal investigator)
Université Félix Houphouët-Boigny
Tel.: +225 0500 8223 / +225 0209 1401
E-mail: couljeanvae@yahoo.fr

The co-principal investigator must immediately notify by phone the local ethical authorities (Comité National d'Éthique de la Recherche (CNER) et au Département de la Pharmacie, du Médicament et des Laboratoires (DPML) about serious adverse events. Following 24 hours, the local co-principal investigator must provide to the study sponsor-investigator further information on the serious adverse event or the unanticipated problem in the form of a written narrative. This should include a copy of a completed SAE form, and any other diagnostic information that will assist the understanding of the event. In exceptional circumstances, a serious adverse event may be reported by telephone. In these cases, a written report must be sent immediately thereafter by fax or e-mail. Names, addresses and telephone for serious adverse event reporting will be included in the trial-specific SAE form. Relevant pages from the CRF may be provided in parallel (e.g., medical history, concomitant medications).

6.4. Safety reporting to Health Authorities and Ethics Committees

The sponsor-investigator will send appropriate safety notifications to Health Authorities in accordance with applicable laws and regulations. Additionally, this information will be provided to 'Ethikkomission Nordwest- und Zentralschweiz' (EKNZ, Switzerland) and the ethics committee and the 'Direction de la Pharmacie, du Médicament et des Laboratoires (DPML)' in Côte d'Ivoire according to national rules. Fatal or life-threatening serious adverse events or SUSARs will be reported within 24 hours after the event followed by a complete report within 7 additional calendar days. The sponsor will suspend dosing of further participants (until review by the PI and study physician(s)), if there is sufficient cause as determined by the investigators including the study physician, which attests that the continuity of the study will jeopardize the health or safety of participants. The study will be suspended pending a new positive decision by the two accredited local aforementioned institutions. The investigator will take care to inform all participants. Participants already enrolled in the study will complete the trial. Other serious adverse events and SUSARs that are not fatal or life-threatening will be filed as soon as possible but no later than 14 days after first knowledge by the sponsor.

7. Statistics

7.1. Definition of primary endpoint

Cure rate is the primary outcome of our study. Since these might be influenced by infection intensity, treatment allocation will be stratified by baseline infection intensity.

7.2. Justification of number of trial subject

Since the existence of a drug effect is well known, the main aim of the study is the elucidation of the nature of the dose–response relationship. A series of simulations was carried out to determine the required sample size. We assumed a true cure rate against hookworm of 5%, 30%, 50%, 70% and 80% (for 0, 200, 400, 600 and 800 mg) and of 5%, 10%, 20%, 30% and 40% against *T. trichiura* and loss to follow up of 5%. We estimated that enrolling 40 participants per arm will be sufficient to predict the dose response curve with a precision (defined as median distance between prediction and confidence band) of about 10 percentage points. The suggested sample size is also in line with the recommendations from Klingenberg et al. 2009 [11]. This sample size is sufficiently high to determine the key PK parameters as well as PK/PD relationships.

7.3. Description of statistical methods

The primary analysis will use the full analysis set (available case population) according to the intention to treat principle defined as all randomized subjects which provide any follow-up data. Only subjects which were negative at baseline and erroneously randomized will be excluded from the analysis. In addition, a per-protocol analysis will be conducted. CRs will be calculated as the percentage of egg-positive participants at baseline who become egg-negative after treatment. EPG will be assessed by adding up the egg counts from the quadruplicate Kato-Katz thick smears and multiplying this number by a factor of six. The ERR will be calculated (ERR = (1-(mean at follow-up/mean at baseline))*100).

Geometric mean egg counts will be calculated for the different treatment arms before and after treatment to assess the corresponding ERRs. Bootstrap resampling method with 5,000 replicates will be used to calculate 95% confidence intervals (CIs) for ERRs point estimates. E_{max} models using the DoseFinding package of the statistical software environment R will be implemented to predict the dose-response curves in terms of CRs and ERRs.

On the basis of the LC-MS/MS measurements, the following PK parameters for plasma will be calculated:

C_{max} maximal plasma concentration

t_{max} time to reach C_{max}

AUC area under the curve, from 0 to 24h and 0 to inf

$T_{1/2}$ elimination half-life

C_{max} and T_{max} will be observed values derived from the plasma concentration time profile. AUC and $T_{1/2}$ will calculated with the software WinNonlin (Version 5.2, Pharsight Corporation, USA) using noncompartmental analysis. The elimination half-life will be estimated by the equation: $T_{1/2} = \ln 2/\lambda$, where λ will be determined by performing a regression of the natural logarithm of the concentration values during the elimination period.

Further PK analysis will be undertaken fitting a structural compartmental PK model with the software NONMEM 7 via nonlinear mixed effects modeling (allowing for both between patient variation and random effects). This model will describe the blood drug levels in time, allowing one to investigate variation between patients in drug levels. In addition, pharmacometric-pharmacodynamic (PKPD) analysis will be undertaken via NONMEM to investigate the drivers of cure and/or burden reduction and potentially any covariates with drug levels.

Adverse events will be evaluated descriptively as the difference of proportion reporting adverse events before and after treatment.

If possible, a sensitivity analysis will be conducted where double infections enrolled in one trial will be included in the other trial.

8. Data management and data quality control

The investigators are responsible for an adequate data quality. Prior to the initiation of the study, a short investigators meeting will be held with the investigators and their study coordinators and a member of the Swiss TPH. This meeting will include a detailed discussion of the protocol, performance of study procedures, CRF completion, specimen collection and diagnostic methods.

8.1. Data collection

The data produced from this research project will fall into the following categories:

1. Eggs counts of hookworm, *Ascaris lumbricoides* and *Trichuris trichiura* found in participants' stool samples using the Kato-Katz technique before (baseline) and after (follow-up) treatment.
2. Personal information such as name, age and gender of trial participants.
3. Anthropometric and clinical characteristics of the trial participants collected using the study's case report form (CRF) such as weight, height, blood pressure, temperature hemoglobin levels, any abnormal medical condition or chronic disease.
4. Number and type of adverse events registered in the CRF and actively probed for 3and 24,hours after treatment. The same data will be collected during the collection of the first sample at follow-up.
5. Pharmacokinetic parameters such as Cmax, AUC or tmax analyzed from dry blood spots or Mitra® at 0, 1, 2, 3, 4, 6, 8, 24 hours post-dosing.

Data from categories 1-4 will be paper-captured and subsequently doubled entered (data entry SOP) into ACCESS data entry masks by two independent people for quality assurance. Both databases will then be cross-checked using the Data Compare utility of EpiInfo; any discrepancies will be corrected by consulting the hard copy. Data entered into ACCESS databases will only be accessible to authorized personnel directly involved with the study by use of a password. Data in category 1 will be saved both in .mdb, .xlsx and .csv. Data in category 2, 3 and 4 will be double-entered using an EpiInfo mask and saved in .mdb, .xlsx and .csv. All categories will be merged into a single master filed saved in .xlsx and .csv. Data will then be analysed as described in section 7. PK parameters derived from analyzing microblood samples will be merged and saved as a .xlsx file. Hard copies of the data such as parasitological sheets and CRFs will remain at IHI. Digital copies along with the databases will be transferred to the Swiss TPH after a Material Transfer Agreement has been signed by both the Swiss TPH and CSRS. All data is expected to not exceed 5GB.

8.2. Ethical, legal and security issues

Screened patients will be listed in a confidential “subject screening log”. Enrolled patients will be listed in a confidential “subject enrolment log” and attributed a unique study number; this document will constitute the only source to decode the pseudonymized data and will only be accessible to the local principal investigator. Electronic data files will be stored on secured network drives with restricted access for study personnel only. Data analysis will be conducted with pseudonymised data and reporting of findings will be fully anonymised; personal data will be coded for data analysis. No names will be published at any time, and published reports will not allow for identification of single subjects. Confidentiality will be ensured throughout the entire research project. All databases will be password secured. None of the investigators declare to have any conflicts of interest.

8.3. Data storage and preservation

After the study has been completed all samples will be destroyed. Data and related material will be preserved for a minimum of 15 years to enable understanding of what was done, how and why, which allow the work to be assessed retrospectively and repeated if necessary. Essential infrastructure such as lockable cabinets for safe storage of hardcopy data will be made available. Storage and backup will be in three places: personal laptops of Jennifer Keiser and Jessica Schulz, Swiss TPH shared server and SWICTHdrive. Archiving conditions will be made strictly confidential by password protection.

9. Duties of the investigator

9.1. Investigator’s confirmation

This trial will be conducted in accordance with the protocol, International Council for Harmonisation of technical requirements for pharmaceuticals for human use harmonized guidelines of Good Clinical Practice E6 (R2) (ICH-GCP) and the current version of the Helsinki Declaration.

All protocol modifications must be documented in writing. A protocol amendment can be initiated by either the Sponsor or any Investigator. The Investigator will provide the reasons for the proposed amendment in writing and will discuss with the Sponsor and the Principal Investigator. Any protocol amendment must be approved and signed by the Sponsor and the Principal Investigator and must be submitted to the appropriate Independent Ethics Committee (IEC) for information and approval, in accordance with local requirements, and to regulatory agencies if required. Approval by IEC must be received before any changes can

be implemented, except for changes necessary to eliminate an immediate hazard to trial subjects, or when the change involves only logistical or administrative aspects of the trial, e.g. change of telephone number(s).

9.2. Damage coverage

A general liability insurance of the Swiss TPH is in place (Winterthur Police Nr. 4746321). A trial insurance will be arranged in Côte d'Ivoire.

9.3. Project management

The trial team will include the PI (Prof. Jennifer Keiser), a trial and data manager (Dr. Jean Coulibaly), a trial statistician (Dr. Jan Hattendorf), as well as a physician (Dr. Yves N'Gbesso) and laboratory technicians. Prof. Jennifer Keiser and Dr. Jean Coulibaly will be responsible for staff management, communication with the collaborative group, recruitment monitoring, data management, safety reporting, analysis, report writing and dissemination of the trial results. Dr. Jean Coulibaly is responsible for supervision of the lab- and field technicians, staff management, recruitment monitoring, supply of the material, contact to the local authorities and participating schools. Dr. Jessica Schulz will be responsible for the pharmacokinetic studies. Dr. Yves Koutouan N'Gbesso will be responsible for all medical aspects of the study, including the decision for inclusion and exclusion of study participants.
The investigator team is responsible for ensuring that the protocol is strictly followed. The investigator should not make any changes without the agreement of the Principal Investigator and the Co-Investigators, except when necessary to eliminate an apparent immediate hazard or danger to a study participant. The investigator will work according to the protocol and GCP. The investigator may take any steps judged necessary to protect the safety of the participants, whether specified in the protocol or not. Any such steps must be documented. During the treatment the records are maintained by the responsible medical doctor. All entries have to be made clearly readable with a pen. The investigator must be thoroughly familiar with the properties, effects and safety of the investigational pharmaceutical product.

10. Ethical considerations

10.1. Independent Ethics Committee (IEC)

The study will be submitted for approval by the institutional research commission of the Swiss TPH, the ethical committees of Switzerland (EKNZ) and Côte d'Ivoire and the Ministry

of Health in Côte d'Ivoire. The study will be undertaken in accordance with the Declaration of Helsinki and good clinical practice (GCP).

10.2. Evaluation of the risk-benefit ratio

Albendazole is a well-known, widely used drug in mass treatment programs against filariasis, and has little and mainly mild adverse events (headache, abdominal pain etc.). All community members enrolled in the study will benefit from a clinical examination and treatment against STHs. All participating subjects remaining positive for *T. trichuria*, hookworm and concomitant STH will be treated with albendazole (according to WHO recommendations).

Biological sampling (stool, blood) is always associated with potential risk of disease transmission. For this reason, only qualified persons with expertise will be involved in sample collection. They will only use sterilized material. Note that, painful swelling and/or numbness of the arm may occasionally occur as a result of blood sampling. These effects are usually transient. This procedure can also occasionally trigger reactions such as mild discomfort and weakness. These reactions are usually benign, short-lived and limited to feelings of weakness, accompanied by sweating. However, biological tests suggested in this work will provide important data on the health of the participants.

10.3. Subject information and consent

All parents or caregivers of eligible children and all adult participants (≥21 years) will be asked to sign a written informed consent sheet. Caregivers must have attained their majority (21 years in Côte d'Ivoire). In case the person is illiterate, an impartial witness that can read and write has to sign the consent and the illiterate participant to give a thumb print. Parents or caregivers will have sufficient time for reflection of their child's participation. Additionally, school-aged children (6-12 years) will be briefed verbally and written assent sought in form of their name written down or, if illiterate, by providing a thumb print. Community meetings will be conducted to explain the purpose and procedures of the study. The parents attending this meeting will receive a small provision to cover their costs for transportation to the study site (~US$ 2). Information sheets are printed in French but will additionally be verbally translated into local languages (i.e. Abbé, Attié, Dioula, Moré) during community meetings. Participation is voluntary and participants have the right to withdraw from the study at any given point in time with no further obligations. Participation itself will not be awarded with compensation. Data collected until date of potential withdrawal will be analyzed and once analysis performed fully anonymized.

10.4. Subject confidentiality

The obtained data will be handled strictly confidentially. Only members of the study team will have access to the data. Personal data will be coded for data analysis. No names will be published at any time, and published reports will not allow for identification of single subjects. Confidentiality will be ensured throughout the entire research project.
The investigators have all been trained in GCPs. None of the investigators declare to have any conflicts of interest.

10.5. Subjects requiring particular protection

This study will be carried out in preschool- and school-aged children, since *T. trichuria* and Hookworm infection occurs often in children; hence, these age groups are at high risk of infection. Pharmacokinetic and dose-finding studies have not been conducted to date in this population. Since PK parameters vary between children and adults these studies cannot be obtained by carrying out the trial on adults. Our trial will pave the way for a safe and effective treatment of STH infections in children.

10.6. Other aspects

We will include one arm of each study cohort treated with placebo. However, this group will be treated with a standard treatment of 400 mg single dose albendazole at the end of the study (3-4 weeks later); hence, this does not cause any medical concern. Patients generally are treated only in yearly intervals to reduce morbidity from chronic infections. A treatment delay of 3-4 weeks is not expected to cause any harm.

11. Quality control and quality assurance

11.1. Monitoring and auditing

We will work with a locally based monitor. He/she will conduct site visits to the investigational facilities for the purpose of monitoring the study. Details will be described in a separate monitoring plan. The investigator will permit them access to study documentation and the clinical supplies dispensing and storage area. Source data/documents will be accessible to monitors and questions will be answered during monitoring. Monitoring observations and findings will be documented and communicated to appropriate study personnel and management. A corrective and preventative action plan will be requested and documented in response to any audit observations. No sponsor initiated audits are foreseen, but audits and inspections may be conducted by the local regulatory authorities or ethics committees. The

Investigator agrees to allow inspectors from regulatory agencies or ethics committees to review records and is encouraged to assist the inspectors in their duties, if requested.

11.2. Access to data, handling of data and samples (data protection), archiving (place, duration) and destruction

Information about study subjects will be kept confidential and managed accordingly (access restricted, password protected, as mentioned in chapter 7.3). A CRF will be completed for each subject enrolled into the clinical study. The investigators will review, and approve each completed CRF. The study CRF is the primary data collection instrument for the study. All data requested on the CRF must be recorded. All missing data must be explained. If a space on the CRF is left blank because the procedure was not done or the question was not asked "N/D" will be entered. If the item is not applicable to the individual case "N/A" will be written. All entries will be printed in black ink. All corrections must be initialed and dated.

All data on parasitology and questionnaires about adverse events and self-reported clinical signs and symptoms will be doubled entered into a database by two independent persons and cross-checked. Discrepancies between data entries will be corrected by consulting the hard copy.

The results of the research study will be published, but subjects' names or identities will not be revealed. Records will remain confidential. To maintain confidentiality, the principal investigator will keep records in locked cabinets and the results of tests will be coded to prevent association with participant's names. Data entered into the ACCESS data entry mask will be accessible only by authorized personnel directly involved with the study and will be encoded. Subject-specific information may be provided to other appropriate medical personnel only with the subject's permission.

After the study has been completed all samples will be destroyed and research data and related material will be kept for a minimum of 15 years to enable understanding of what was done, how and why, which allow the work to be assessed retrospectively and repeated if necessary.

11.3. Data entered directly in the Case Report Form (CRF) – definition of source data

Source Data are the clinical findings and observations, laboratory data maintained at the study site. Source data are contained in source documents. Local authorities are allowed to access the source data. Data will be entered directly onto the case report forms. The case report form (CRF) is considered a ***source document***. All CRFs will be kept for at least 15 years.

The study site will retain a copy of the CRF to ensure that local collaborators can provide access to the ***source documents*** to a monitor, auditor, or regulatory agency.

11.4. Data and safety monitoring board (WHO) / data monitoring committee (EU/FDA)

In our study no DSMB will be established, since we work with well-known drugs in a small sample size and using a single dose treatment.

11.5. Study Documents: Translations - Reference language

- Protocol: Master document in English, all further language versions are translations thereof
- CRF: Master document in English, all further language versions are translations thereof
- ICF: no English version available; master document in French, all further language versions are translations thereof

12. Dissemination of results and publication

The final results of this study will be published in a scientific journal and presented at scientific conferences. All results from this investigation are considered confidential and shall not be made available to any third part by any member of the investigating team before publication.

13. References

1. Jourdan PM, Lamberton PHL, Fenwick A, Addiss DG: Soil-transmitted helminth infections. *Lancet* 2017.

2. Murray CJ, et al: Global, regional, and national disability-adjusted life years (DALYs) for 306 diseases and injuries and healthy life expectancy (HALE) for 188 countries, 1990-2013: quantifying the epidemiological transition. *Lancet* 2015, **386**(10009):2145-2191.

3. Bethony J, Brooker S, Albonico M, Geiger SM, Loukas A, Diemert D, Hotez PJ: Soil-transmitted helminth infections: ascariasis, trichuriasis, and hookworm. *Lancet* 2006, **367**(9521):1521-1532.

4. WHO: Prevention and control of schistosomiasis and soil-transmitted helminthiasis: report of a WHO expert committee, vol. No. 912. Geneva: World Health Organization; 2002.

5. Moser W, Schindler C, Keiser J: Efficacy of recommended drugs against soil transmitted helminths: systematic review and network meta-analysis. *BMJ (Clinical research ed)* 2017, **358**:j4307.

6. Horton J: Albendazole: A review of anthelmintic efficacy and safety in humans. *Parasitology* 2000, **121**:S113-S132.

7. Pengsaa K, Limkittikul K, Pojjaroen-anant C, Lapphra K, Sirivichayakul C, Wisetsing P, Nantha-aree P, Chanthavanich P: Single-dose therapy for giardiasis in school-age children. *Southeast Asian J Trop Med Public Health* 2002, **33**(4):711-717.

8. Katz N, Chaves A, Pellegrino J: A simple device for quantitative stool thick-smear technique in schistosomiasis mansoni. *Rev Inst Med Trop Sao Paulo* 1972, **14**(6):397-400.

9. Speich B, Ali SM, Ame SM, Albonico M, Utzinger J, Keiser J: Quality control in the diagnosis of Trichuris trichiura and Ascaris lumbricoides using the Kato-Katz technique: experience from three randomised controlled trials. *Parasit Vectors* 2015, **8**(1):82.

10. Speich B, Ame SM, Ali SM, Alles R, Huwyler J, Hattendorf J, Utzinger J, Albonico M, Keiser J: Oxantel pamoate-albendazole for Trichuris trichiura infection. *The New England journal of medicine* 2014, **370**(7):610-620.

11. Klingenberg B: Proof of concept and dose estimation with binary responses under model uncertainty. *Statistics in medicine* 2009, **28**(2):274-292.
